# Development and precision evaluation of a robotic system for oral implant surgery using personalized digital guides and optical spatial positioning technology

**DOI:** 10.1371/journal.pone.0319054

**Published:** 2025-04-29

**Authors:** Heqiang Tian, Yurui Yin, Xiang Zhang, Jiezhong Tan, Junqiang Liu

**Affiliations:** College of Mechanical and Electronic Engineering, Shandong University of Science and Technology, Qingdao, China; High Point University, UNITED STATES OF AMERICA

## Abstract

Oral implant surgery demands a high level of precision and expertise, making the integration of robotic assistance an optimal solution. This study introduces an innovative dental implant robotic system designed to enhance accuracy during cavity preparation by combining robotic technology, optical spatial positioning, and personalized digital implant guides. The system employs an EC66 six-degree-of-freedom robotic arm, integrated with digital implant guides and optical navigation technology. A customized implant guide mapping device was developed, fabricated, and validated for its guiding accuracy through meticulous registration and measurement processes. The robotic system’s coordinate systems were thoroughly analyzed, and hand-eye calibration, along with tool calibration, was implemented to ensure synchronized spatial transformations and complete spatial information registration.The robotic system demonstrated superior angular precision in cavity preparation, significantly reducing angular deviation compared to traditional manual methods (4.17 ± 0.28° vs. 5.23 ± 0.10°). However, no significant differences were found in top surface deviation (1.09 ± 0.37 mm vs. 1.43 ± 0.06 mm) or root surface deviation (1.49 ± 0.57 mm vs. 2.57 ± 0.10 mm) between the robotic and manual approaches. These results indicate that while the robotic system excels in angular control, surface deviations in the top and root regions remain comparable to those achieved through manual methods. The system effectively reduces human error, particularly in angular precision, ensuring greater directional control during the procedure. Despite these advancements, further improvements are needed in model and template printing precision, as well as optimization of calibration methods, to minimize residual errors and enhance overall accuracy.This study presents a novel and reproducible approach for assessing the accuracy of implant guides and robotic positioning systems. By showcasing the potential of robotic systems to improve surgical precision and outcomes in dental implantology, this research offers valuable insights for future clinical applications and technological advancements in the field.

## 1. Introduction

With the continual advancements in modern medical technology, oral implant surgery has emerged as a widely adopted and efficacious method for the restoration of missing teeth. In the context of an aging population, the demand for oral implant procedures has markedly increased. Dental implants, often termed the “third tooth of mankind,” offer restorative outcomes that closely emulate natural teeth in function, structure, and aesthetics. This resemblance has led a growing number of patients with tooth loss to prefer dental implants as their method of restoration. Owing to their non-destructive nature, dental implants are esteemed by the dental community as the optimal solution for tooth replacement [1].

Oral implant surgery is a sophisticated procedure, with cavity preparation serving as the pivotal step. This socket preparation necessitates a high degree of expertise and operational precision from the clinician. The constrained space within the oral cavity, coupled with the occlusion of hard and soft tissues, frequently impedes dentists from completing cavity preparation under direct visualization. Minor operational errors or deviations in precision can negatively impact long-term functional and aesthetic outcomes and may even compromise critical anatomical structures surrounding the tooth, resulting in irreparable harm to the patient [2].

Among various surgical procedures, oral implant surgery exhibits a significant degree of adaptability to robotic assistance due to the rigid nature of the alveolar bone, which aligns well with traditional robotic work objects. This adaptability renders robot-assisted cavity preparation an ideal alternative to manual techniques. With the ongoing development and application of digital technologies in oral implantation, an increasing number of dentists are opting to utilize computer-assisted tools for socket preparation. Currently, static digital implant guides and dynamic real-time surgical navigation are the two most commonly employed tools for cavity preparation by dental professionals.

Numerous scholars, both domestically and internationally, have conducted extensive research in the domain of oral implant surgical robots. The Yomi system, developed by Neocis in the United States, is the world’s first FDA-approved oral implant robot. It integrates CBCT imaging and haptic feedback with user-friendly operation and human-machine interaction, incorporating artificial intelligence and machine learning technologies into surgical planning and real-time decision-making to achieve high-precision oral implant surgery [3]. The Robodent system, developed collaboratively by Germany and Israel, utilizes a high-precision robotic arm and three-dimensional imaging technology to achieve accurate implant positioning, demonstrating favorable clinical outcomes [4]. The oral implant robot developed jointly by Tokyo Medical and Dental University and several high-tech companies minimizes surgical trauma and accelerates postoperative recovery through high-precision robotic arm control and an advanced navigation system [5]. Boesecke et al. translated implant program information from the patient’s coordinate system to the surgical robot’s coordinate system. Using a preoperative program, they employed a robot-held drill guide to determine the initial drilling site, direction, and depth, thereby assisting the dentist in performing the surgery and directly applying the preoperatively developed treatment plan to the patient [6]. Pires et al. constructed an oral implant robotic system comprising an ABB industrial robot, a data acquisition board, strain gauges for stress/strain assessment, and a force/torque transducer placed on the robot’s wrist. They investigated the effect of the number of implants and their placement locations on stress/strain distribution [7]. Wilmes et al. developed an oral implant robotic system using an RX60 robot as the main body, which includes a robotic arm, an angle sensor, and a torque/force sensor. Their study provides significant guidance for the clinical application of micro-implants [8,9].

Sun et al. developed an image-guided dental implant robotic system that reconstructs a three-dimensional model of the patient using CBCT images. This system completes implant design by recognizing the datum attached to the patient’s jawbone and recording its coordinates in a virtual (image) coordinate system [10]. Yu et al. integrated robotics with an image navigation system, enabling surgeons to operate the robotic arm remotely. The manipulator applies an adjustment force when approaching the target, facilitating precise convergence to the target and avoiding collisions, thereby significantly enhancing the accuracy of dental implant surgery [11]. Li et al. developed a novel robotic manipulation system specifically for dental implantation, which substantially enhances the stiffness, force capacity, and accuracy of the manipulator [12,13]. Researchers at the Air Force Medical University and Beijing University of Aeronautics and Astronautics have developed and launched the world’s first “fully autonomous” dental implant surgical robotic system. This system employs optical positioning technology to obtain the position information of a device worn in the patient’s mouth. The robot integrates this target position information with the preoperative implant program to prepare the socket for the patient [14,15].

Intraoperative alignment techniques for oral implant robots are critical to ensuring the accuracy and safety of dynamic real-time surgical navigation. Current alignment methods include three-dimensional reconstruction based on CT or CBCT images, optical tracking systems with marker alignment, point cloud alignment technology based on surface scanning, and alignment based on dental implant guides. Image alignment techniques utilize CT and CBCT images to achieve high-precision matching between preoperative image data and the surgical site through precise image processing and alignment algorithms. Multimodal data fusion techniques comprehensively leverage various image and sensor data to enhance the accuracy and reliability of spatial registration [16,17]. Optical tracking systems capture and update the positions of surgical tools and patient anatomy in real time by establishing positioning markers and reference frames [18,19]. By integrating patient CBCT images with CAD/CAM technology, the digital design of implant guides can be completed and fabricated through three-dimensional printing. Image alignment, optical tracking, and mechanical alignment are employed to achieve high-precision oral implant surgery. Digital implant guides are capable of accurately controlling the direction, angle, and depth of dental drills, thereby maximizing the final surgical outcomes [20,21]. These methods incorporate advanced image processing algorithms and machine learning techniques to achieve high-precision anatomical structure matching. Despite some clinical successes, intraoperative alignment still faces challenges such as the matching accuracy of complex anatomical structures, real-time performance, and uncertainties arising from patient movement [22].

Registered calibration of oral implant robotic systems involves mapping the robot coordinate system, navigator coordinate system, and tool coordinate system to ensure that the robotic end tool operates accurately based on target information. Hand-eye calibration and tool calibration are pivotal techniques to ensure surgical accuracy. Hand-eye calibration aligns the motion of the robot arm with the coordinate system of the vision sensor and is categorized into two modes: “eye outside the hand” and “eye on the hand.” Common methods include optical tracking based on multiple viewpoints, the use of calibration boards, and laser calibration. Methods for resolving the hand-eye transformation include Tsai’s method [23], the double quaternion method [24], and nonlinear optimization methods [25]. Tool calibration ensures that the position and orientation of the drilling tool at the end of the robot are precise. Common methods for tool calibration involve using precision mechanical and optical measurement equipment [26].

In this study, we aim to enhance the precision of cavity preparation in robotic oral implant surgery by integrating robotics, optical spatial positioning technology, and personalized digital implant guides. We investigate a dental implant robotic system based on a dental implant guide mapping device to develop methods for robotic cavity preparation. Specifically, this study constructs a dental implant robot system, designs and fabricates a personalized dental implant guide mapping device for the surgical area, analyzes the guiding accuracy of the implant guide, registers the spatial information, and validates the accuracy of the robotic system. Additionally, we conduct dental implant robot cavity preparation experiments to verify the feasibility of the dental implant guide-based robotic cavity preparation method. This study offers a novel approach to cavity preparation for oral implant surgery, possessing significant research importance and practical value.

## 2. Materials and methods

### 2.1 Construction of the oral implant robot system

The digital implant guide plate is integrated with a spatial mapping device, enabling the registration of implant plan data within the coordinate system of the spatial mapping device. By obtaining the position of the spatial mapping device’s coordinate system, which is worn on the patient’s teeth, the navigation system indirectly acquires the implant plan information through matrix transformation. This transformation converts the data into the coordinate system of the robot’s base, allowing the robot to autonomously perform cavity preparation without the need for manual intervention.

**Fig 1:**
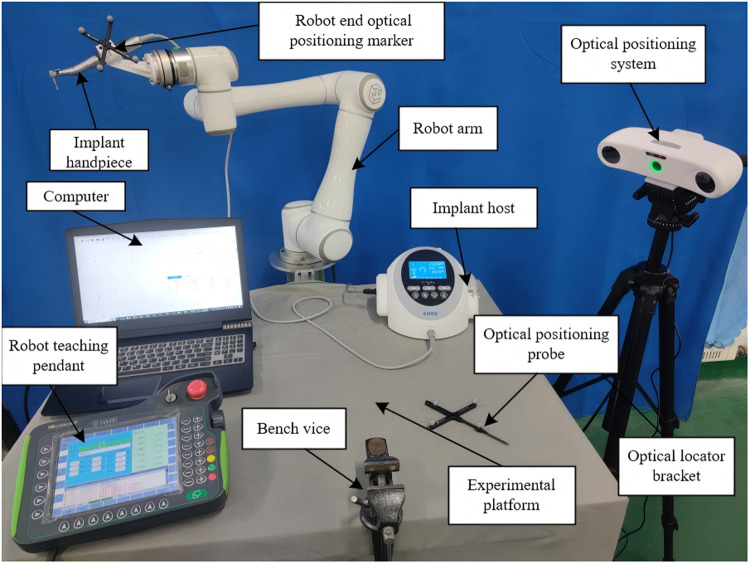
Oral implant robot system.

In consideration of the specific requirements inherent to oral implant surgery, the navigation system operates within a fixed field of view. As a result, the “Eyes-to-Hand” configuration is adopted in the construction of the oral implant robotic system. This system consists of several key components, including a computer, a robotic arm, an implant machine, an optical positioning system, and a digital implant guide mapping device, as illustrated in Fig 1.

The system setup involves several critical steps: First, a six-degree-of-freedom (6-DoF) robot is mounted on the experimental platform, with the implant machine connected to the robot’s end flange via an end connecting device. The computer and implant machine are then positioned on the experimental platform to facilitate operation, while a bench vice secures the patient’s tooth-mucosa model. The optical locator is carefully positioned to ensure that the experimental area remains within its field of vision. Finally, communication connections are established among all system components. These steps collectively enable the system to efficiently and accurately translate and apply the preoperatively designed implant plan to the surgical procedure, enhancing the precision and safety of cavity preparation.

Given the constraints imposed by the limited space within the oral cavity and the stringent precision requirements of implant surgery, the selected robot must be both lightweight and capable of achieving high positioning accuracy, as outlined in Table 1. The chosen C-Sailor implant system is equipped with a high-performance brushless motor, which offers the ability to preset torque and speed, and supports both forward and reverse rotations, along with adjustable speed ratios. This motor is renowned for its stability, low noise production, and minimal heat generation, with its technical specifications provided in Table 2.

**Table 1:** Robot-related parameters.

Model	EC66 six-degree-of-freedom robot
**Dead weight**	17.5 kg
**Payload**	6 kg
**Working radius**	914mm
**Protection level**	IP54
**Range of joints**	± 360 °
**Repeat positioning accuracy**	± 0.03 mm
**Tool maximum speed**	2.8 m/s

**Table 2:** C-sailor planter technical parameters.

Model	Yusen planter (C-sailor)
**Output Power**	120W
**Brushless motor speed**	300–50,000 rpm
**Torque**	5.0 to 55N.cm (20:1)
**Infusion pump flow (Maximum)**	150 ml/min
**Power supply**	AC100V/60Hz

The AP-LIT-100 optical positioning system employs FPGA technology to enhance the stability and synchronization of image acquisition, reduce image noise, and enable precise localization and real-time tracking of surgical tools within a three-dimensional space. With a high positioning accuracy of up to 0.2 mm, low latency, compact size, and a wide measurement range, this system is particularly well-suited for the confined and challenging environments typical of surgical procedures.

By leveraging these advanced technologies, the system ensures enhanced accuracy and reliability in oral implant surgeries. This approach effectively addresses the challenges of working within the restricted operational space of the oral cavity while meeting the demanding requirements for precision and real-time performance essential in surgical practice.

### 2.2 Design and fabrication of the dental implant guide plate mapping device

#### 2.2.1 Full-information oral digital model reconstruction and optimization.

**Fig 2:**
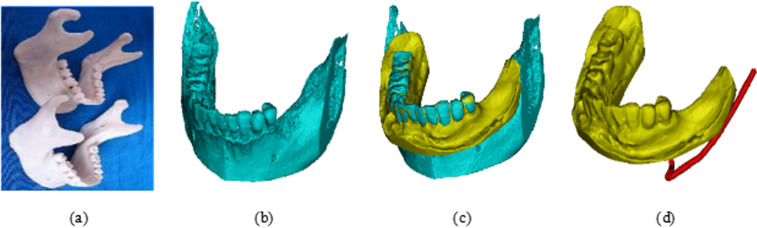
Comprehensive digital model of the artificial jaw model: (a) Artificial jaw model; (b) 3D digital model of the jaw; (c) Registered model; (d) Nerve line depiction.

The structural information of the artificial jaw model (Fig 2(a)) was obtained using cone-beam CT (CBCT), while the digital model of the tooth-mucosal surface morphology was captured using three-dimensional laser scanning technology. The CBCT image data were imported into Mimics medical software for threshold segmentation, mask editing, and 3D reconstruction, resulting in a digital model of the missing dentition and jawbone, as shown in Fig 2(b).To reconstruct the digital model of the oral cavity, the 3D model of the missing teeth and jaws was set as the base image, while the digital model of the teeth and mucosa was designated as the floating image. Paired corner and inflection points were selected on both images to perform manual multi-point alignment. The “Global Alignment” function was then used to optimize the alignment, generating the 3D digital model of the artificial jaw model, as depicted in Fig 2(c). Lastly, the nerve line adjacent to the missing tooth was drawn using the software, based on the structural information of the nerve canal within the jawbone. After the nerve line was defined, the jawbone model was hidden, completing the reconstruction of the fully informative oral digital model of the artificial jaw model, as shown in Fig 2(d).

The undercut filling process on the dental model using 3-matic software involves importing the model and identifying areas with surface pits and gaps between adjacent teeth. The Surface Selection tool is used to identify these areas, and the Smooth or Fill Surface functions are applied to eliminate irregularities. The Repair Undercut tool is then employed to address any undercut regions, ensuring a smooth surface. After filling, the Remesh function is used to adjust the mesh and prevent distortion, while Surface Analysis verifies surface smoothness. Finally, Boolean Operations are applied to merge and refine the filled areas, and the model is exported in STL format for further use. This process eliminates undercuts and optimizes the model for personalized dental guide plate design.

#### 2.2.2 Implant program design.

**Fig 3:**
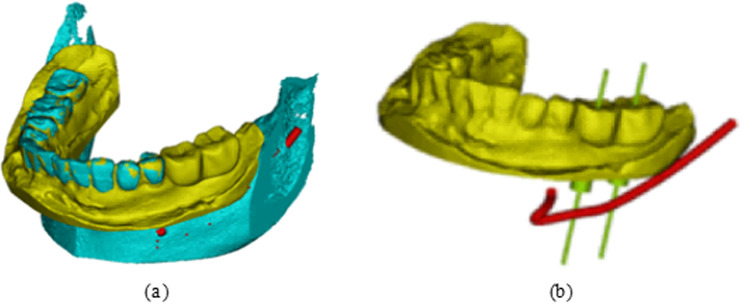
Virtual implant program design: (a) Diagnostic tooth placement; (b) Virtual implant model.

The virtual implant is created by designing a cylinder within Mimics software and adjusting its position to complete the implant program, guided by the expertise of the implant scheme designer. To implement the implant-based restoration, a pre-designed diagnostic tooth model was introduced into the digital model of the fully informative oral structure of the artificial jaw model. The position of the diagnostic tooth model served as a reference for the direction and placement of each implant, as shown in Fig 3(a). In the designed implant scheme, the virtual implants positioned at tooth locations 36 and 37 are depicted in Fig 3(b).

#### 2.2.3 Structural design of the dental implant guide plate.

**Fig 4:**
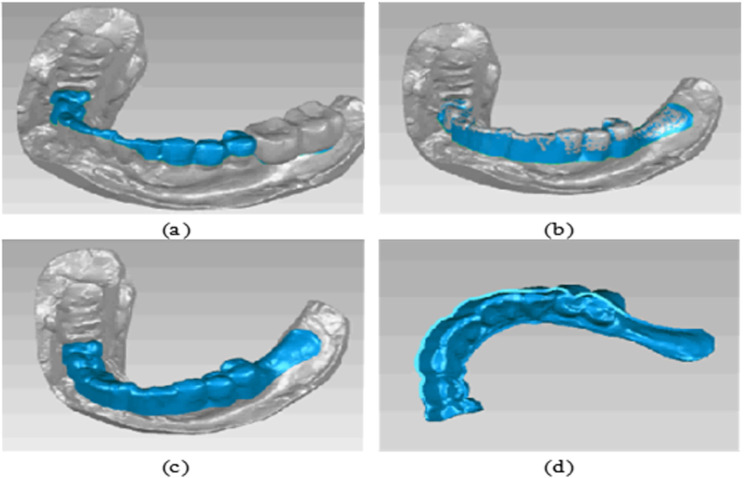
Guide plate substrate design: (a) Selected regions; (b) Edge extension; (c) Thickened surfaces; (d) Substrate model.

**Fig 5:**
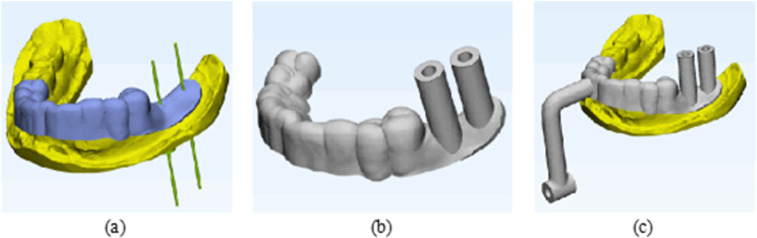
Guide plate mapping connection design: (a) Position relationship between guide plate and implant; (b) Finalized digital model; (c) Connection device.

To design the implant guide plate, Geomagic Wrap software was utilized to select the fixed area of the guide plate on the surface of the processed tooth-mucosa model. The boundary curve and surface trimming of the model were adjusted, followed by the application of the shell extraction function to thicken the model, creating the preliminary guide plate substrate, as shown in Fig 4. The tooth-mucosa model and the virtual implant model were then exported from Mimics software and imported into 3-matic software. The positional relationship between the implant guide plate matrix and the virtual implant is illustrated in Fig 5(a). Using the axis of each implant as a reference, analysis cylinders were created, and various operations such as wrapping, trimming, and Boolean subtraction were applied to design the personalized guide part of the implant guide plate. Boolean operations were performed with the guide plate matrix to generate a complete digital model of the implant guide plate, as shown in Fig 5(b).

The designed implant guide plate and the STL format file of the connecting rod, created in SolidWorks, were imported into 3-matic software. Boolean operations and adjustments to the connecting pose were carried out to construct a complete digital model of the spatial mapping connecting device, as shown in Fig 5(c). Throughout this process, Geomagic Wrap software was used to refine the fixed area of the guide plate by adjusting boundary curves and surface trimming of the filled dental-mucosa model. The shell extraction function was again applied to ensure proper thickening, providing a foundation for further processing. Various operations, including wrapping, trimming, and Boolean commands, were carried out to integrate the implant guide plate matrix with the personalized guide design, ensuring the seamless construction of the final digital model.

#### 2.2.4 Fabrication of the dental implant guide mapping device.

**Fig 6:**
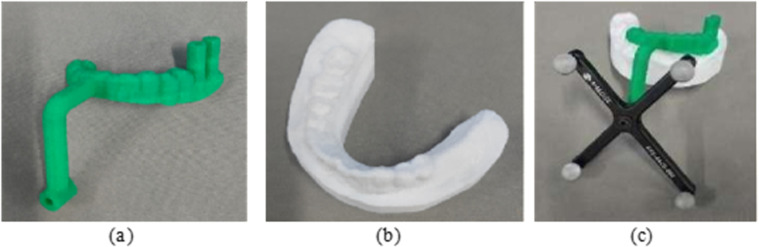
Fabrication and assembly of the implant guide mapping device: (a) Printed guide; (b) 3D-printed tooth-mucosa model; (c) Final assembly.

The Allcct Printer 300 was used to print the dental implant guide mapping connector. The personalized dental implant guide mapping device was imported into the Allcct Printer design software in STL format. The software processed the cross-sectional information from the file and decomposed the 3D model into layer-by-layer cross-sections. The printing parameters were adjusted within the software and saved as a G-code file, which was then imported into the 3D printer to complete production. The printed implant guide is smooth, continuous, strong, and resistant to deformation, as shown in Fig 6(a). Additionally, the 3D model of the tooth-mucosa was thickened at the base using Geomagic Wrap software and printed in 3D to serve as a substitute for the artificial jaw model during experimentation, as shown in Fig 6(b). Finally, the personalized dental implant guide mapping device was tested on the tooth-mucosa model to assess the fit and stability of the assembly, as illustrated in Fig 6(c).

### 2.3 Spatial information registration of the the oral implant robot system

#### 2.3.1 Establishment of the spatial coordinate system.

**Fig 7:**
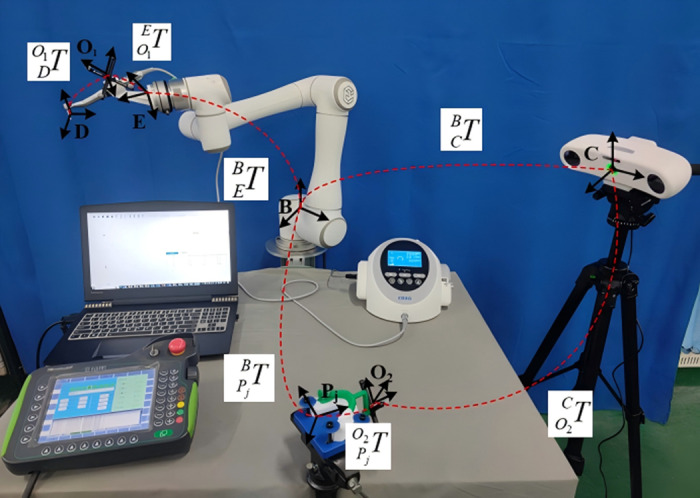
Coordinate diagram of the oral implant robot system.

To accurately represent the various components of the robot system, multiple 3D Cartesian coordinate systems are established: Robot base coordinate system $B_{XYZ}$ , Robot end flange coordinate system $E_{XYZ}$, Robot end optical positioning marker coordinate system $O_{1XYZ}$, Implantable handpiece drill pin coordinate system $D_{XYZ}$, Optical positioning system coordinate system $C_{XYZ}$, and Implant guide plate mapping device coordinate system (Patient coordinate system) $O_{2XYZ}$, as illustrated in Fig 7.

The transformation matrix TEB between the robot end flange coordinate system $E_{XYZ}$ and the robot base coordinate system $B_{XYZ}$ is derived using the robot’s forward kinematics. Similarly, the transformation matrix TOiC between the optical positioning marker coordinate system ${\left(O_{XYZ}\right)}_{i}$ (i=1,2) and the optical positioning system coordinate system $C_{XYZ}$ is obtained in real-time through the optical positioning system. Preoperatively, the position transformation matrix TPjO2 of the implant coordinate system ${\left(P_{XYZ}\right)}_{j}$ (denoted as *j* for the tooth position number) within the implant guide plate mapping device coordinate system $O_{2XYZ}$ is acquired using an optical positioning probe under the optical positioning system coordinate system. Additionally, it is essential to determine the transformation matrix TCB between the optical localization tracker coordinate system $C_{XYZ}$ and the robot base coordinate system $B_{XYZ}$, as well as the transformation matrix TDE between the drill pin coordinate system $D_{XYZ}$ and the robot end flange coordinate system $E_{XYZ}$. These transformation matrices TCB and TDE are obtained through hand-eye calibration and tool calibration, respectively.

#### 2.3.2 Hand-eye calibration.

To calibrate the hand-eye system, the optical positioning marker is securely fixed on the robot’s end effector. The robot end effector is then moved multiple times to various positions, and the corresponding position of the optical positioning marker is recorded in the optical positioning system’s coordinate system. Simultaneously, the position data of the robot’s end effector is captured in the robot base coordinate system. These data points are used to establish a mathematical model, and a specific algorithm is applied to solve the hand-eye transformation matrix, completing the calibration process.

In the “eye outside the hand” configuration of the hand-eye system, the optical positioning marker on the robot’s end effector remains fixed relative to the end connection device. Consequently, the transformation matrix between the end flange coordinate system and the optical positioning marker coordinate system is a constant matrix during the movement of the robot’s end effector. The following kinematic equations are established to describe these relationships:


TO1E=TBEiTCBTO1Ci=TBEi+1TCBTO1Ci+1 (1)

Where:

TBEi represents the transformation relation between the robot’s end-flange coordinate system and the base coordinate system during the *i-*th motion. This can be obtained through the robot’s forward kinematics.

TCB denotes the transformation relationship between the optical positioning system coordinate system and the robot base coordinate system, which is the matrix to be solved.

TO1Ci refers to the transformation relationship between the optical positioning system coordinate system and the robot end optical positioning marker coordinate system during the *i-*th motion, which can be obtained in real-time from the optical localization device.

By rearranging the above equation, the relationship can be expressed as:


TBEi+1−1TBEiTCB=TCBTO1Ci+1TO1Ci−1 (2)

By noting that A=TBEi+1−1TBEi, B=TO1Ci+1TO1Ci−1 and X=TCB, the above equation can be written as:


AX=XB (3)

Where:

A represents the transformation relation between two neighboring robot end positions (i.e., the chi-square transformation matrix between two different recording points in the robot base coordinate system). This is derived from the robot’s forward kinematics.

B denotes the motion relationship between two neighboring positioning markers (i.e., the chi-square transformation matrix between two different recording points in the optical positioning system coordinate system). This is calculated based on data acquired by the optical positioning system.

*X* represents the robot hand-eye calibration matrix.

To solve the hand-eye calibration equation, the Tsai-Lenz two-step calibration method [2,3] is employed. This method linearizes the nonlinear problem by utilizing rotation matrix properties. *A*, *B* and *X* in Eq. (3) are 4×4 chi-square transformation matrices, and the equation is simplified for computational clarity as follows:


RBEitBEi01RCBtCE01=RCBtCB01RO1CitO1Ci01 (4)

The equation is then transformed into the hand-eye calibration fundamental form:


RBEi⋅RCB=RCB⋅RO1CiRBEi−I⋅tCB=RCB⋅tO1Ci−tBEi (5)

The Rodrigues Transform is applied to express the rotation matrices as corresponding rotation vectors, where:


rO1Ci=rodriguesRO1CirBEi=rodriguesRBEi (6)

The rotation vector is normalized as:


NrO1Ci=rO1Ci/rO1CiNrBEi=rBEi/rBEi (7)

Solve for the hand-eye initial rotation vector PCB':


SkewpO1Ci+pBEi⋅PCB'=pBEi−pO1Ci (8)

Recording *N* sets of data will provide *N*-1 of the above equations for a least squares solution.

Solve for the rotation vector PCB:


PCB=2PCB'1+PCB'2 (9)

Solve for the rotation matrix RCB:


RCB=1−pCB22I+12PCBPCB'+4−PCB2⋅SkewPCB (10)

#### 2.3.3 Calibration of surgical instruments.

The six-point calibration method is employed to calibrate the tool coordinate system, ensuring precise alignment and operation of the tool during dental implant surgery. This process involves two key components: the position calibration of the Tool Center Point (TCP) and the pose calibration of the Tool Coordinate Frame (TCF). These steps define the tool’s origin and orientation relative to the robot’s end coordinate system, enabling accurate and reliable robot-assisted procedures.

In the TCP position calibration, the robot’s end tool is manually moved to touch a reference point in four distinct postures. The first three points are arbitrarily oriented, while at the fourth point, the tool tip must be perpendicular to the worktable plane. To ensure this vertical alignment, the tool’s posture is precisely controlled through the robot’s inverse kinematics, aligning the tool tip with the normal direction of the worktable. This action guarantees that the tool tip contacts the reference point perpendicularly. The vertical alignment is critical because any deviation from this alignment can affect the accuracy of subsequent operations, particularly in needle tip calibration. If the tool tip is misaligned, it may introduce errors that compromise the precision of delicate procedures such as dental implant surgeries. Proper vertical alignment ensures both the direction and position of the tool are accurate, which is essential for precise needle tip calibration. Any deviation in vertical alignment can lead to errors in calculating the needle tip’s position, ultimately impacting the overall accuracy of the surgical procedure.

For the TCF pose calibration, the robot’s end tool is manually adjusted along the X and Z axes. Starting from the fourth point, the tool tip is moved a predefined distance along the X-axis to record the fifth point, followed by a similar movement along the Z-axis to record the sixth point. These movements provide the positional data necessary to calculate the pose matrix of the tool relative to the robot’s end coordinate system. While the Z-axis is used as the reference direction in the tool coordinate system, it does not necessarily align perfectly with the direction of the drill. The Z-axis typically aligns with the main axis of the tool during operation but is not identical to the drill’s cutting direction. The drill’s cutting direction refers to the rotation axis of the drill, while the Z-axis serves as the reference for ensuring proper tool alignment in space, which is critical for accurate positioning and tool performance during surgery.

**Fig 8:**
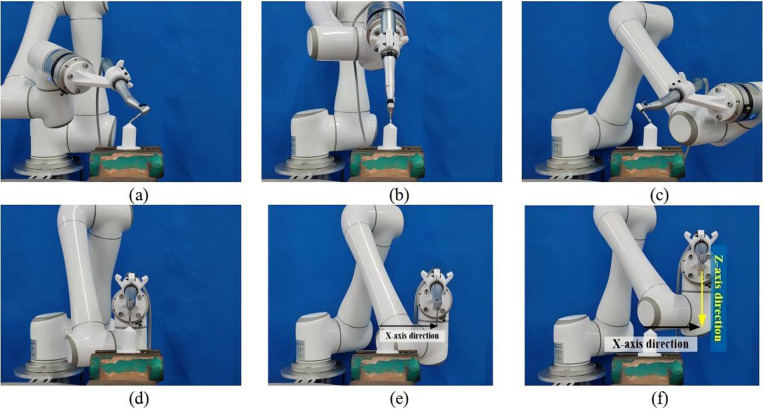
Six-point method for tool calibration: respectively, show the six position points during the tool calibration: (a) Position point 1; (b) Position point 2; (c) Position point 3;(d) Position point 4; (e) Position point 5; (f) Position point 6.

This six-point calibration method ensures the precise alignment of the tool’s coordinate frame with the robot’s coordinate system, which is essential for achieving reliable, accurate operations in dental implant surgery. The method mitigates operational errors, enhances repeatability, and ensures the tool’s positional and directional accuracy. The calibration procedure is visually illustrated in Fig 8, which shows the six distinct calibration points. This comprehensive approach guarantees that both the TCP and TCF are precisely calibrated, ensuring the accuracy and reliability of the robot-assisted surgical procedure.

(1) Position calibration of TCP

During the TCP calibration process, the transformation matrix TDB under the tool coordinate system to the robot base coordinate system for the *i-*th (*i*=1, 2, 3, 4) calibration point is shown in Eq. (11):


TDBi=TEBiTDE (11)

A chunked form expansion of the above equation yields:


RDBiPDBi01=REBiPEBi01RDEPDE01=REBiRDEREBiPDE+PEBi01 (12)

Since the elements of (1, 2) on both sides of the equation correspond equally, it follows that:


PDBi=REBiPDE+PEBi (13)

Since the origin of the tool coordinate system in the above posture is fixed at a constant position under the robot base coordinate system, i.e., PDBi (i=1,2,3,4) is a definite value, the following relation holds:


REB1PDE+PEB1=REB2PDE+PEB2REB2PDE+PEB2=REB3PDE+PEB3REB3PDE+PEB3=REB4PDE+PEB4 (14)

In the above equation PDE, which contains three elements and contains 3(*n*-1) equations, the coefficient matrix is a 3(*n*-1) × 3 matrix, which is a column full rank matrix when *n* ≥ 3. The above equation, *n*=4, is an incompatible equation and can only be solved as a least squares solution, i.e.,:

The above equation is shifted and transformed into matrix form as shown in Eq. (15):


REB1−REB2REB2−REB3REB3−REB4︸A⋅PDE=PEB2−PEB1PEB3−PEB2PEB4−PEB3︸b (15)

In the above equation PDE, which contains three elements and contains 3(*n*-1) equations, the coefficient matrix is a 3(*n*-1) × 3 matrix, which is a column full rank matrix when *n* ≥ 3. The above equation, *n*=4, is an incompatible equation and can only be solved as a least squares solution, i.e.,:


PDE=A+⋅b (16)

The first term on the right hand side of the above equation is the additive generalized inverse of the coefficient matrix, which is a column-full rank matrix, so its additive generalized inverse satisfies the following relation:


$$A^{+}={\left(A^{T}\cdot A\right)}^{-1}\cdot A^{T}$$ (17)

Substituting Eq. (17) into Eq. (16) yields:


PDE=REB1−REB2REB2−REB3REB3−REB4T⋅REB1−REB2REB2−REB3REB3−REB4−1⋅REB1−REB2REB2−REB3REB3−REB4T⋅PEB2−PEB1PEB3−PEB2PEB4−PEB3 (18)

Eq. REBi can be found from the following equation:


REBxyz(γ,β,α)=R(ZB,α)R(YB,β)R(XB,γ)=cα−sα0sαcα0001cβ0sβ010−sβ0cβ1000cγ−sγ0sγcγ=cαcβcαsβsγ−sαcγcαsβcγ+sαsγsαcβsαsβsγ−cαcγsαsβcγ−cαsγ−sβcβsγcβcγ (19)

Substitute position data of the calibration point $P_{1},P_{2},P_{3},P_{4}$ into Eq. (19) and convert it into the form of a pose matrix, then substitute it into Eq. (18) to calculate the position calibration result PDE of the end effector tip in the robot’s end effector coordinate system.

(2) Pose calibration of TCF

After completing the tool calibration, the pose matrix of the end effector needs to be calculated. The tool pose calibration is performed using the Z/X direction method. During this process, the robot’s end effector pose is kept unchanged, and the pose information of three calibration points $P_{4},P_{5},P_{6}$ is read. The vector relationship between the calibration points $P_{4}$ and $P_{5}$ (+X direction) is equivalent to the direction vector along the +X axis of the actual tool coordinate system. Therefore, the axial vector along the X-axis of the tool coordinate system can be obtained, as shown in Eq.(20).


X=PEB5x−PEB4xPEB5y−PEB4yPEB5z−PEB4z (20)

Similarly, the axial vector along the +Z axis of the actual tool coordinate system can be obtained, as shown in Eq.(21).


Z=PEB6x−PEB5xPEB6y−PEB5yPEB6z−PEB5z (21)

The *Y* axial vectors are determined using the right-hand rule, as shown in Eq.(22).


Y=Z×X (22)

To ensure the correctness of the coordinate system vectors, the condition in Eq.(23) must be satisfied.


Z=X×Y (23)

After calculating the axial vectors for each axis from Eq.s (20), (22), and (23), they are normalized to obtain the pose matrix RDB of the tool coordinate system relative to the base coordinate system. Then, by multiplying the inverse of the rotation matrix REB−1 of the end effector coordinate system relative to the base coordinate system, the rotation matrix RDE of the tool coordinate system relative to the end flange coordinate system can be obtained, as shown in Eq.(24).


RDE=(REB)−1RDB (24)

From Eq.(19) and defining the variables RDE=r11r12r13r21r22r23r31r32r33, the rotation angles $\theta_{Z}=arc\tan2(r_{21}/r_{11})$, $\theta_{Y}=arc\sin(-r_{31})$ and $\theta_{X}=arc\tan2(r_{32}/r_{33})$ can then be calculated.

#### 2.3.4 Acquisition of the actual position in the drill pin coordinate system of the implant handpieces.

**Fig 9:**
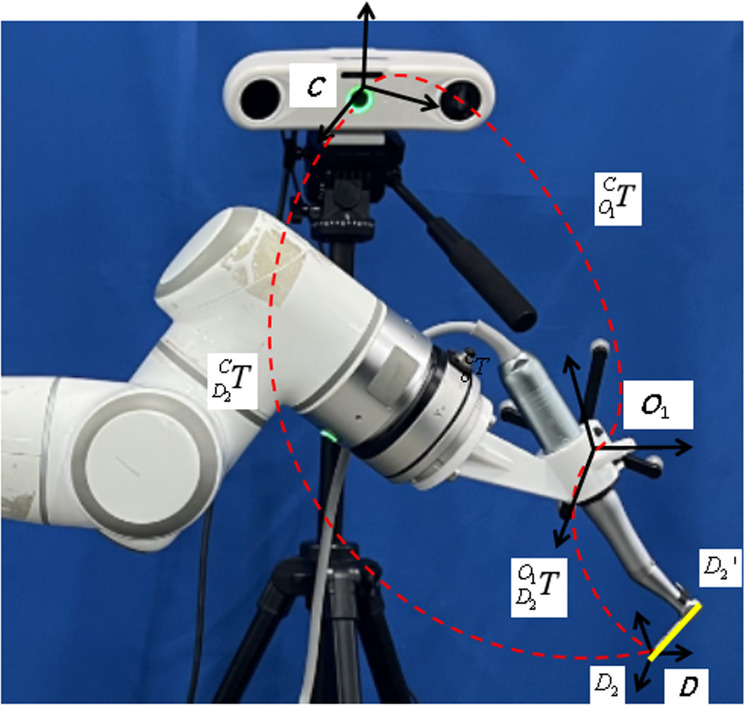
The actual position information acquisition in the drill pin coordinate system of the implant handpieces.

In order to enable the optical positioning system to obtain the position information of the implant handpieces drill pin coordinate system in real time, it is necessary to complete the calibration of the transformation matrix TDO1 between the robot end optical positioning mark coordinate system $O_{1XYZ}$ and the implant handpieces drilling needle coordinate system $D_{XYZ}$, as shown in Fig 9. Using the coordinate system of optical positioning system as the intermediate coordinate system, the position information of the robot end optical positioning mark coordinate system TO1C and the position information of the implant handpieces drill pin coordinate system TDC under the optical positioning system are obtained respectively, and then the target matrix TDO1 is obtained through matrix transformation.

The position transformation relationship TO1C between the optical positioning system coordinate system and the robot end optical positioning mark coordinate system can be calculated from the position data acquired by the optical positioning system; in order to acquire the position transformation relationship between the optical positioning system coordinate system and the implant handpieces drill pin coordinate system, we firstly select the feature point and define its coordinate system again, and then establish its coordinate system according to the position information of the acquired feature point under the coordinate system of the optical positioning system, and then obtain the transformation matrix.

(a) Finding the transformation relationship TO1C between the robot end optical positioning marker and the optical positioning system coordinate system.

The position information of the coordinate system $O_{1XYZ}$ of the robot end optical positioning marker under the optical positioning system $C_{xyz}$ can be read out in real time, and based on the position information, the transformation matrix TO1C between the coordinate system of the robot end optical positioning marker $O_{1xyz}$ and that of the optical positioning system $C_{xyz}$ is calculated.

(b) Finding transformation relationship TD2C between the optical positioning system and the implant handpiece drill pin coordinate system.

The position information of the tip $D_{2}$ and root D2' of the implant handpiece drill pin obtained by the probe under the optical positioning system coordinate system $O_{1XYZ}$. In order to define the implantable handpiece drill pin coordinate system, three non-collinear points are needed, and the position information of each point obtained by taking the origin of the robot end optical positioning marker coordinate system $O_{1xyz}$ as the third point.

Set the tip end point *D* of the drill pin of the implant handpiece as the origin of the coordinate system, the vector $\vec{H}$ direction of the point D2' pointing to the point $D_{2}$ is parallel to the Z-axis of the coordinate system,take the plane in which D2D2'→ and $\vec{D_{2}O_{1}}$ are located as the YOZ plane of the coordinate system, take the normal vector $\vec{P}$ of this plane to be parallel to the *X* axis of the coordinate system, and then calculate the direction vector $\vec{Q}$ parallel to the *Y* axis based on the two direction vectors that have been determined, correct the direction of the vectors and unitize them, and then unite the origins to obtain the position matrix TD2C of the coordinate system $D_{2XYZ}$ under the optical positioning coordinate system $C_{XYZ}$.

(c) Solving the constant transformation matrix TD2O1 between the coordinate system of the robot end optical positioning marker and the coordinate system of the implant handpiece drill pin.

TD2O1=(TO1C)−1TD2C (25)

#### 2.3.5 Registration of the implant-guided mapping device position information.

(1) Registration method

**Fig 10:**
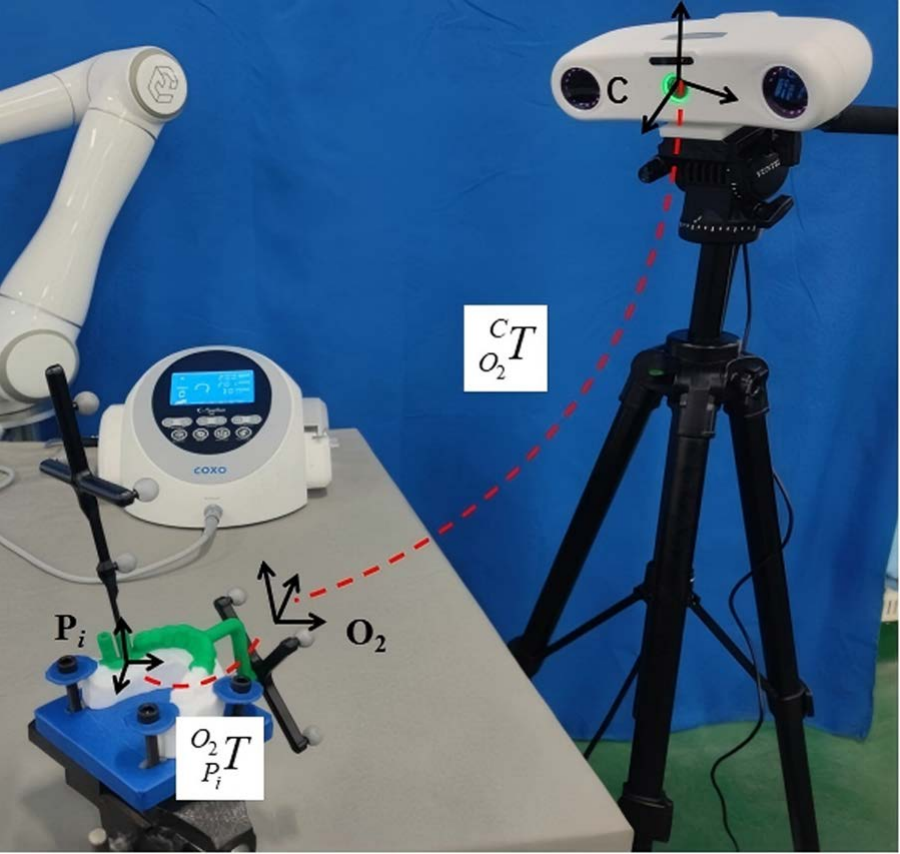
Implant program information registration of the dental implant guide mapping device.

The registration of dental implant guide plate mapping device implant program information, the purpose is to register and record the virtual implant position program information into the coordinate system of the positioning marker connected with it, obtain the spatial position information of the positioning marker through the optical positioning system in real time, and obtain the information of the virtual implant cavity preparation program under the coordinate system of the optical positioning system through the matrix transformations, which is an important link in the whole system. The whole registration process is carried out within the field of view of the optical positioning space, the registration scene is shown in Fig 10, and the registration method is as follow:

(a) Determine the initial drilling position in the coordinate system $O_{2XYZ}$:The initial implant position *P* is set at approximately one millimetre from the mucosa of the oral dentition, and the initial implant position is determined in conjunction with the optical positioning system by means of the optical positioning probe that has already been calibrated and recorded in the coordinate system $O_{2XYZ}$ in the form of a matrix transformation; the initial implantation position is determined by means of the optical positioning system.
(b) Determine the direction vector of the implant guide plate mapping device guiding hole axis in the coordinate system $O_{2XYZ}$: According to the principle of determining a vector of two points, in the implant guide plate guiding hole up to the end of the guiding cylinder to select another point *Q* (to ensure that it does not detach from the guiding hole), determine the position of the point in the optical positioning system, and then obtain the direction vector of the implant axis by converting and associating the position of the two points in the coordinate system $O_{2XYZ}$.
(c) Determine the depth of the cavity to be prepared: In the Mimics design software, the distance from the bottom of each virtual implant in its axial direction to the mucosal surface, i.e., the depth of the cavity to be prepared, can be obtained using the measurement tool.

(2) Registration process

The registration process of the virtual implant initial implant position information is completed under the normal acquisition of the optical localization tracker, and the position information of each point under the coordinate system of the optical localization tracker is recorded, as shown in Table 3. During the registration process, the position of the dental implant guide mapping device and the optical positioning tracker is fixed, so the position information of the device coordinate system under the optical positioning tracker coordinate system $C_{xyz}$ is unchanged, and the position information of the positioning marker can be captured using optical positioning($T_{x}$, $T_{y}$, $T_{z}$, $R_{x}$, $R_{y}$, $R_{z}$).

**Table 3:** Position information of each point under the optical positioning system.

Missing tooth position	Initial drilling point *P*/mm			Guide cylinder end point *Q*/mm		
36	${\left(P_{36}^{\;C}\right)}_{x}$	${\left(P_{36}^{\;C}\right)}_{y}$	${\left(P_{36}^{\;C}\right)}_{z}$	${\left(Q_{36}^{\;C}\right)}_{x}$	${\left(Q_{36}^{\;C}\right)}_{y}$	${\left(Q_{36}^{\;C}\right)}_{z}$
37	${\left(P_{37}^{\;C}\right)}_{x}$	${\left(P_{37}^{\;C}\right)}_{y}$	${\left(P_{37}^{\;C}\right)}_{z}$	${\left(Q_{37}^{\;C}\right)}_{x}$	${\left(Q_{37}^{\;C}\right)}_{y}$	${\left(Q_{37}^{\;C}\right)}_{z}$

By substituting the localization marker position information into Eq. (19), the constant transformation matrix TO2C of the spatial localization marker coordinate system $O_{2xyz}$ in the operative area under the optical localization system coordinate system $C_{xyz}$ can be calculated.

The positions of the points obtained in the optical locator coordinate system $C_{xyz}$, as shown in Table 3, are transformed into the localization marker coordinate system $O_{2xyz}$ through the calculation in Eq. (26). The results of this transformation are presented in Table 4.

**Table 4:** Position information of each point in the coordinate system. $O_{2xyz}$

Missing tooth position	Initial drilling point *P*/mm			Guide cylinder end point *Q*/mm		
36	${\left(P_{36}^{\;O_{2}}\right)}_{x}$	${\left(P_{36}^{\;O_{2}}\right)}_{y}$	${\left(P_{36}^{\;O_{2}}\right)}_{z}$	${\left(Q_{36}^{\;O_{2}}\right)}_{x}$	${\left(Q_{36}^{\;O_{2}}\right)}_{y}$	${\left(Q_{36}^{\;O_{2}}\right)}_{z}$
37	${\left(P_{37}^{\;O_{2}}\right)}_{x}$	${\left(P_{37}^{\;O_{2}}\right)}_{y}$	${\left(P_{37}^{\;O_{2}}\right)}_{z}$	${\left(Q_{37}^{\;O_{2}}\right)}_{x}$	${\left(Q_{37}^{\;O_{2}}\right)}_{y}$	${\left(Q_{37}^{\;O_{2}}\right)}_{z}$


PO2=(TO2C)−1PC (26)

The virtual implant coordinate system is established with the initial implantation site defined as the origin of the implant coordinate system. The vector $\vec{PQ}$ (denoted as $\vec{W}$) from the initial implantation point *P* to the target point *Q* is aligned parallel to the *Z*-axis of the implant coordinate system. The *XOZ* plane of the implant is defined by the plane formed by $\vec{PQ}$ and $\vec{PO_{2}}$, ensuring that the orientation of the implant coordinate system matches that of the end-effector tool coordinate system. This alignment prevents collisions between the implant tool and natural teeth or surrounding tissues during the implantation procedure.

The normal vector $\vec{V}$ of the defined plane is set parallel to the *Y*-axis of the implant coordinate system. Based on the known directional vectors $\vec{W}$ and $\vec{V}$, the directional vector $\vec{U}$, which is parallel to the X-axis, is calculated. All vectors are corrected and normalized to ensure the accuracy and consistency of the attitude information.

By combining the normalized attitude information with the coordinates of the initial implantation site, the transformation matrix of the implant coordinate system in the $O_{2XYZ}$ coordinate system is constructed. Using this method, the vectors such as ${\vec{U}}_{36}$, ${\vec{V}}_{36}$ and ${\vec{W}}_{36}$ for the implant at position 36 can be calculated.The transformation matrix TP36O2 between the localization marker coordinate system $O_{2XYZ}$ and the implant coordinate system $P_{36XYZ}$ can be found by unitizing ${\vec{U}}_{36}$, ${\vec{V}}_{36}$ and ${\vec{W}}_{36}$ and associating the origin of the coordinate system.The conversion matrix TP37O2 for implant position 37 can be derived in the same way.

#### 2.3.6 Implant pose information acquisition in the robot base coordinate system.

**Fig 11:**
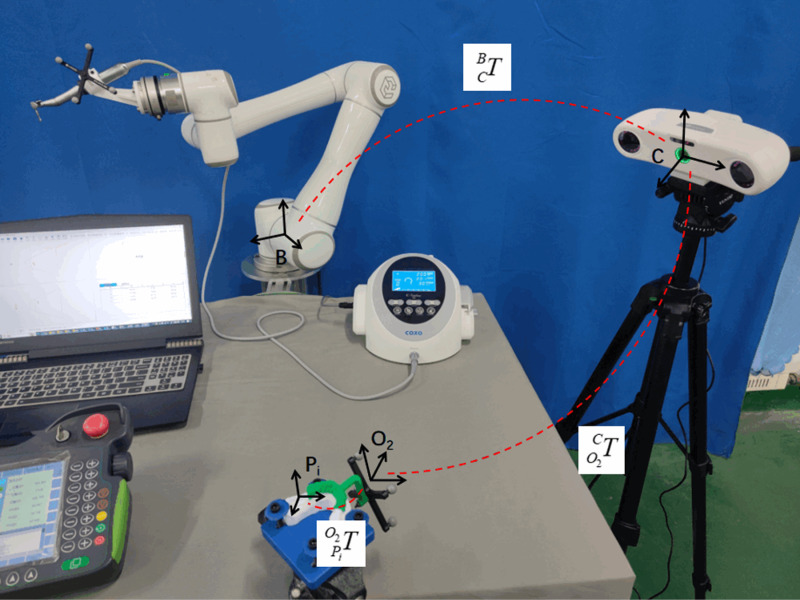
Implant posture information acquisition in the robot base coordinate system.

The guiding component of the registered guide plate mapping device was modified and fitted to the mandibular tooth-mucosa model. The virtual implant position information, relative to the robot base coordinate system, was derived from the positional data of the mapping device obtained via an optical positioning system. The coordinate system transformation relationships for the experimental setup are depicted in Fig 11.

From the analysis of the above figure, it can be seen that the initial implant position in the robot base coordinate system can be obtained by matrix transformation, and the transformation relationship is shown in the following equation:


TPB=TCBTO2CTPiO2 (27)

Where TCB has been derived by the hand-eye calibration method (the hand-eye system positional relationship has been fixed) and TO2C can be acquired in real time by the optical positioning system, and TPiO2 is the implant position registration information that has been completed in the previous section.

The pose information($T_{x}$, $T_{y}$, $T_{z}$, $R_{x}$, $R_{y}$, $R_{z}$) of coordinate system $O_{2XYZ}$ obtained using the optical tracking system after wearing the model is transformed into matrix TO2C form using Eq. (19).

The positional information of each virtual implant in the robot base coordinate system $B_{xyz}$ can be obtained as follows:


TP36B=TCBTO2CTP36O2TP37B=TCBTO2CTP37O2 (28)

### 2.4 Cavity preparation experiment of the oral implant robot

#### 2.4.1 Experimental design.

This study employs a parallel control experiment design, consisting of two groups: an experimental group utilizing a dental implant robot system for cavity preparation and a control group where an experienced oral implant surgeon performs cavity preparation using traditional surgical tools. The design is elaborated on the following five aspects: experimental objectives, subjects, platforms and equipment, grouping scheme, and data measurement and analysis methods.

(1) Experimental objectives

The primary aim of this study is to verify the accuracy and stability of the dental implant robot system during cavity preparation on a simulated mandibular tooth-mucosa model. By comparing the robotic system with traditional surgical methods, the study seeks to evaluate the potential advantages of the robotic system in reducing errors, enhancing precision, and optimizing implantation outcomes. These findings aim to provide experimental evidence supporting the clinical application of robotic-assisted dental implant systems.

(2) Experimental objects

Synthetic mandibular tooth-mucosa models and personalized dental implant template mapping devices were created, as described in Section 2.2. The models were generated using CT and oral scanning data, with high-precision and moderately rigid PLA (polylactic acid) material to ensure consistency and mechanical performance repeatability. Each model includes two target cavity sites (e.g., positions 36 and 37) designed with precise cavity depths and angles to simulate clinical conditions. The virtual implant plans were developed using Mimics and 3-matic software, ensuring consistent initial conditions across all experimental subjects.

(3) Experimental platform and equipment

**Fig 12:**
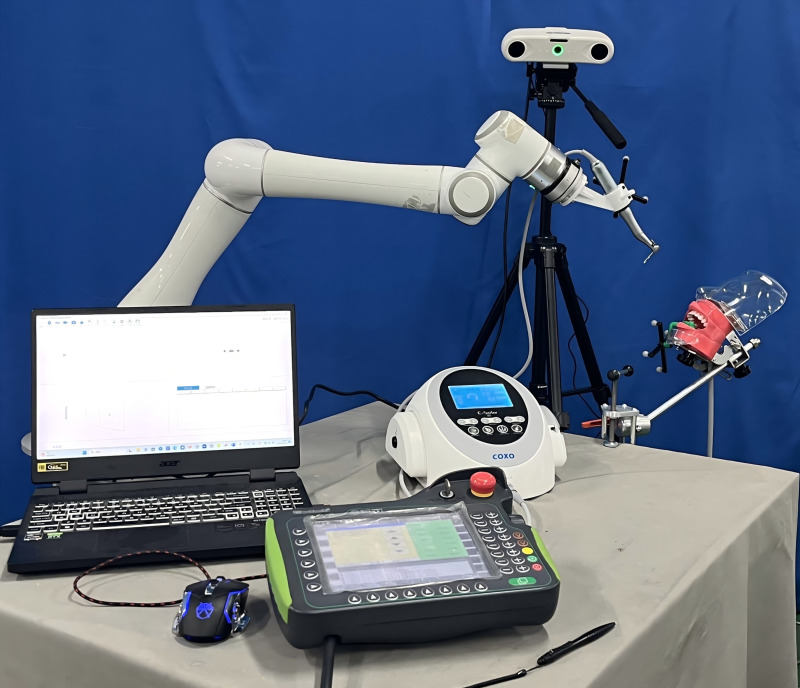
Oral implant robot cavity preparation experiment scenario.

In the experiment, the personalized dental implant template mapping device needs to be worn on the experimental model’s mandibular tooth-mucosa model. The positioning during surgery will be completed using the implant template mapping device. The mandibular tooth-mucosa model is fixed on the experimental platform using an oral model clamp. The experimental setup is shown in Fig 12.

The experimental equipment includes a dental implant robot system based on the EC66 six-degree-of-freedom robot, which offers a repeat positioning accuracy of 0.05 mm. This system is integrated via an interface with the Yusen implant machine and standard surgical tools to perform cavity preparation in the experimental group. Additionally, an optical positioning tracking system is used to monitor the spatial pose of the template mapping device and the target cavity in real time, achieving a positioning accuracy of 0.1 mm. The Runyes dental optical scanner from BlueWild Medical is employed for high-precision post-operative scanning of the cavity shape, with sub-millimeter resolution. Finally, a master control computer runs various experimental design-related software, including Mimics, 3-matic, Geomagic Wrap, and SPSS, to support the analysis and execution of the experiment.

(4) Grouping and experimental variables

The grouping setup for the experiment includes an Experimental Group (Robot Group), where cavity preparation is performed using the dental implant robot system in combination with a personalized template mapping device for precise guidance and positioning. The Control Group (Manual Group) involves cavity preparation conducted by an oral implant surgeon with over 5 years of clinical experience using traditional surgical guides. The experimental variables include the independent variable, which is the method of cavity preparation (robotic vs. manual traditional), and the dependent variables, which are the cavity preparation error indicators, specifically top deviation, root deviation, and angle deviation. Each group will complete 5 independent experiments, each including 2 cavity preparation sites (positions 36 and 37), resulting in a total of 10 cavity data points for statistical analysis.

(5) Data measurement and statistical analysis

The error measurement methods for this study include post-operative scanning, where the surface model STL data of the cavity after preparation is obtained using registration blocks and an optical scanner. For model registration and error analysis, the actual prepared cavity model is imported into 3-matic software and registered with the virtual implant plan model, using the tooth-mucosa model as a reference to measure top deviation, root deviation, and angle deviation. Statistical analysis methods involve data preprocessing, with normality tests performed in SPSS using the Shapiro-Wilk or Kolmogorov-Smirnov tests. For significance testing, if the data follows a normal distribution, independent sample t-tests will be used to compare differences between the two groups. If the data does not follow a normal distribution, non-parametric tests (Mann-Whitney U test) will be employed. The significance level is set at *P*<0.05.

#### 2.4.2 Experimental procedure.

The experimental procedure includes virtual implant planning registration, acquisition of implant posture information, cavity preparation operation, and post-operative data collection and analysis. The specific steps are as follows:

(1) Virtual implant plan registration

**Fig 13:**
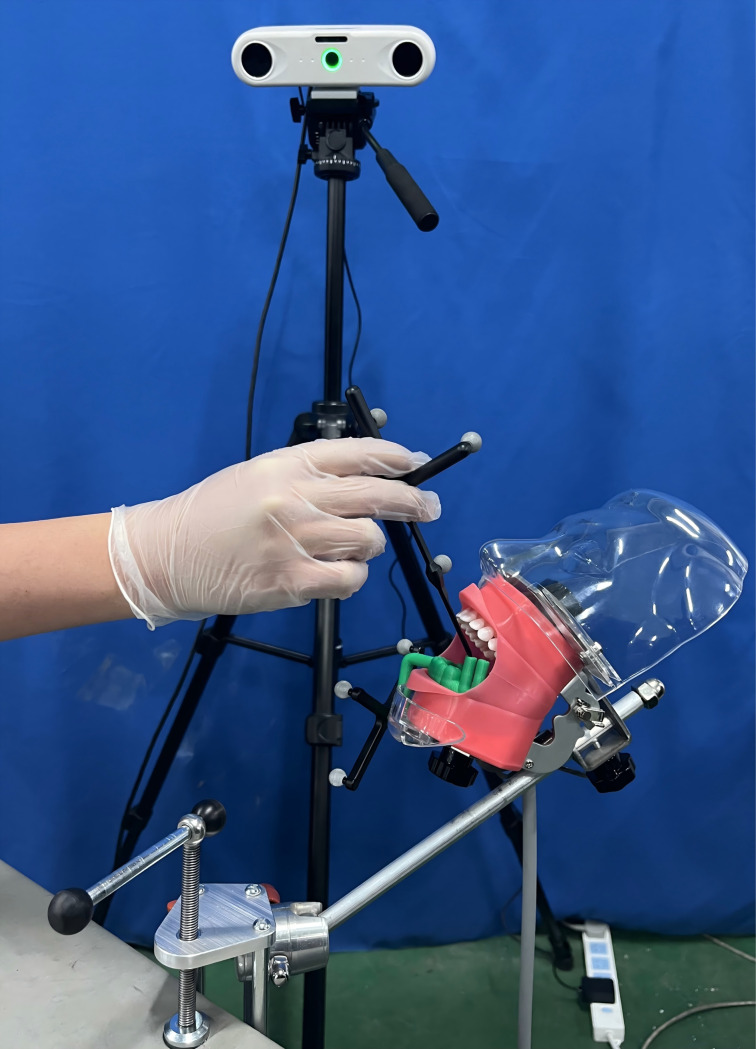
Registration scenario of pose information for the dental implant guide plate mapping device.

Following the registration method outlined in Section 2.3.5, the optical positioning system is used to complete the pose registration of the personalized guide plate mapping device, ensuring accurate alignment of the virtual implant plan with the actual surgical coordinate system. The transformation matrix TP36O2 and TP37O2 between the positioning marker coordinate system $O_{2XYZ}$ and the implant coordinate system is calculated, and the registration process is shown in Fig 13.

(2) Acquisition of implant posture information

**Fig 14:**
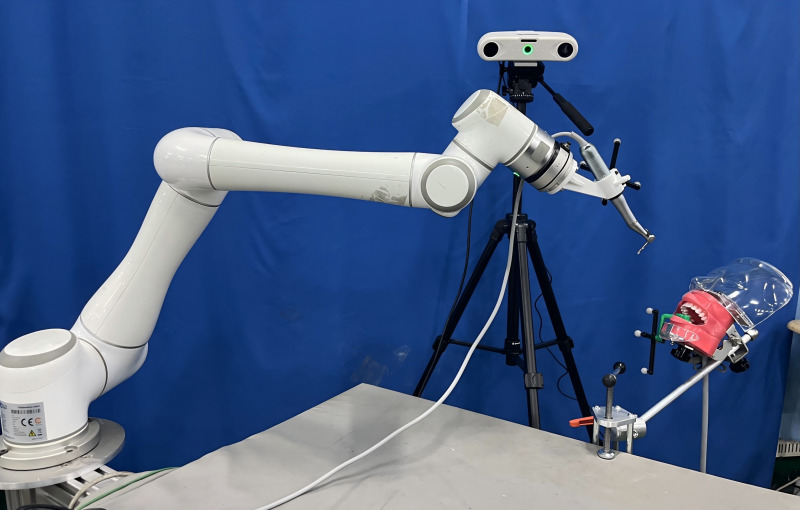
Acquisition scenario of implant posture information in the robot base coordinate system.

Following the method in Section 2.3.6, the guiding part of the registered guide plate mapping device is trimmed and removed, then placed on the mandibular tooth-mucosa model of the experimental setup. The optical positioning tracking system is used to acquire the pose information of the mapping device, and the posture information TP36B and TP37B of each virtual implant in the robot base coordinate system is calculated. The registration process is shown in Fig 14.

(3) Cavity preparation operation for Experimental Group

The robot system performs precise cavity preparation according to the feeding path of the virtual implant plan. Multi-stage feeding and retraction are used to effectively reduce thermal damage and drill wear. The specific steps for the robot cavity preparation are formulated as follows:

(a) Calculate the start-stop position: Determine the start-stop positions for each virtual implant by obtaining the necessary data from the implant program.
(b) Set lifting and pulling actions: Perform the lifting and pulling actions, return the drill to its initial position after reaching the target depth, pause for 3 seconds, and proceed with the next increment until the predetermined depth is achieved.
(c) Repeat for different cavities and drill changes:For cases requiring changes in dental drills or preparation of different cavities, repeat steps 1 and 2 as necessary.

**Fig 15:**
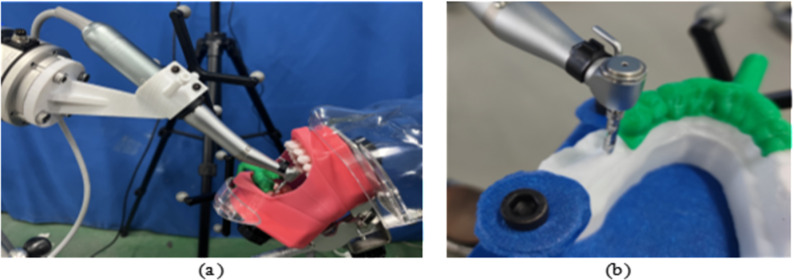
Robotic and manual cavity preparation scenario: (a) Robot cavity preparation; (b) Manual cavity preparation.

**Fig 16:**
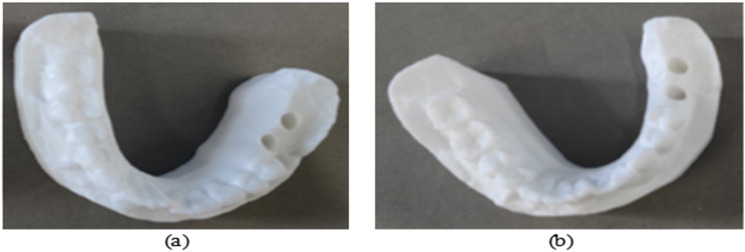
Comparison of robotic and manual cavity preparation results: (a) Robotic cavity; (b) Manual cavity.

Subsequently, the motion program is written, and the surgical tool at the end of the robot is manipulated to complete the coarse positioning. The positioning program is then initiated to perform fine positioning and verification. Once verified, the implant machine system is activated, and the cavity preparation feeding program begins, initiating the drilling process. The robotic cavity preparation scene is illustrated in Fig 15(a).To ensure the accuracy of the experimental results, three sets of experiments were performed, and the tooth-mucosa model after robotic cavity preparation is shown in Fig 16(a).

(4) Cavity preparation operation for Control Group

Manual cavity preparation is performed by an oral implant surgeon with over 5 years of clinical experience using traditional surgical guides. The surgeon endeavors to strictly follow the virtual implant plan during the operation, ensuring that the depth, angle, and position of the cavity align with the design requirements. During preparation, the surgeon relies on their clinical expertise to adjust the feeding path while minimizing operational errors and potential damage to surrounding tissues, achieving the best comparative experimental results. Key operational steps are recorded by the experiment supervisor to ensure the standardization of the control group procedures and the comparability of experimental data. The manual cavity preparation scenario is shown in Fig 15(b), and the tooth-mucosa model after manual cavity preparation is shown in Fig 16(b).

(5) Post-operative data acquisition and error analysis

After the cavity preparation is completed, a designed registration block is inserted into the actual cavity to ensure a tight fit with the cavity shape. A high-precision optical scanner is then used to acquire the 3D surface data (in STL format) of the experimental model. The scanned data is imported into the 3-matic software, where the tooth-mucosa model serves as the reference for model registration and error measurement. By comparing the actual cavity with the virtual implant plan, values for top deviation, root deviation, and angle deviation are recorded separately for the experimental and control groups. This ensures the accuracy and completeness of the data, providing a reliable basis for subsequent statistical analysis.

## 3. Analysis and discussion of results

### 3.1 Accuracy analysis of the dental implant guide plate mapping device

**Fig 17:**
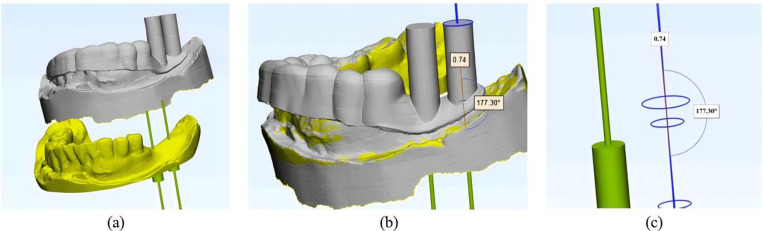
Model registration and error measurement: (a) Model registration; (b) Relative position after registration; (c) Axis error measurement.

In this study, the guiding accuracy of dental implant guides was measured by first importing the scanned assembly digital model and the original implant planning scheme model file into 3-matic software for registration. During the registration process, the guide plate and the axis of the virtual implant were identified as features. The vertical distance and angular error between the axis of the guide plate and the virtual implant were then measured using the software’s measuring tools, as illustrated in Fig 17.

To ensure the reliability of the results, five sets of experiments were conducted, all using the same tooth-mucosa model. For each experiment, the implant guide was assembled onto the model, and the vertical distance and angular error between the axis of the guide portion and the axis of the virtual implant were recorded. These measurements were made based on the method described above, which involved scanning the surface of the assembly model using a dental optical scanner, followed by the registration of the digital model with the original implant planning model. The error measurement process for the guide plate and virtual implant axes was performed five times in total. The results are shown in Table 5.

**Table 5:** Distance and angle error between the axis of the guide plate and virtual implant.

Numbers	Tooth number	Axis distance error/mm	Axis angle error/°
1	36	0.84	2.36
	37	0.74	2.70
2	36	0.83	2.37
	37	0.73	2.71
3	36	0.85	2.34
	37	0.74	2.70
4	36	0.83	2.36
	37	0.72	2.71
5	36	0.86	2.33
	37	0.73	2.69

Based on the data in the table above, it was calculated that the distance and angle error between the axis of the guide plate and virtual implant in the five groups of experiments at positions 36 and 37 were 0.84±0.02mm and 0.73±0.01mm, respectively. The average deviation of the angle was 2.35±0.02° and 2.70±0.01°. These results were derived from five measurements performed on the same tooth-mucosa model, ensuring the consistency and reliability of the data. The findings demonstrate that the designed implant guide exhibits high guiding accuracy and good repeatable seating performance, which meets the guiding accuracy requirements for dental implant surgery.

### 3.2 Verification of spatial registration positioning accuracy for the oral implant robot system

**Fig 18:**
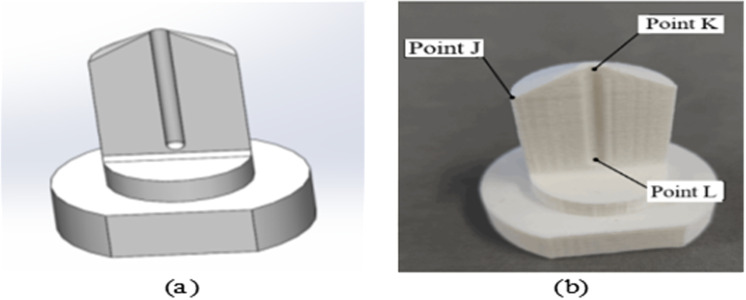
Positioning block design and fabrication: (a) Three-dimensional model of positioning block; (b) Production of positioning blocks and definition of the position of each point.

The positioning accuracy of the oral implant robot system can be measured by comparing and analyzing the theoretical position information obtained from the optical positioning system’s coordinate system with the actual position information in terms of position error. To facilitate the determination of multiple sets of position information in space, this study designs a positioning block. The characteristic points of this positioning block are used to define its own coordinate system and obtain the position information in the optical positioning space. Since determining the coordinate system in space requires three non-collinear points, the design and production of the localization block, along with the definition of the characteristic points, are illustrated in Fig 18.

To ensure the accuracy and credibility of the experimental results, ten groups of experiments were conducted to verify the overall positioning accuracy of the system. Below is the specific analysis and calculation process for one group.

(1) Obtain the position information of the positioning block: In the optical positioning space, the positioning block is fixed in a bench vise. Using a probe, a set of feature point information is collected, as shown in Table 6.

**Table 6:** Position information of each feature point of positioning block under optical positioning system.

Feature points	Feature point coordinates/mm
$J_{1}$	124.592, 159.090, 1041.283
$K_{1}$	115.203, 123.550, 1038.578
$L_{1}$	117.036, 146.187, 1050.910

Define the coordinate system of the positioning block and find the transformation relationship TK1C: set the point $K_{1}$ as the origin of the coordinate system, the direction of the vector $\vec{G}$ pointing to the point $K_{1}$ from the point $L_{1}$ is parallel to the *Z*-axis of the coordinate system, and take the plane where $\vec{KJ}$ and $\vec{KL}$ are located as the *XOZ* plane of the coordinate system, and take the normal vector $\vec{F}$ of this plane parallel to the *Y* axis of the coordinate system, then according to the two direction vectors identified, the direction vector $\vec{E}$ which is parallel to the *X* axis is computed, and then correct the direction of the vectors and carry out the unitary, and then add the rotational matrix by associating the information of the origin position to the rotation matrix. The chi-square matrix of the coordinate system under the optical positioning system coordinate system can be obtained by augmenting the matrix, and the calculation steps are as follows.

Determine a vector by two points:


$$\{\begin{array}{c} \vec{G}=\vec{K_{1}L_{1}}={\left[\begin{array}{ccc} 1.833 & 22.637 & 12.332 \end{array}\right]}^{T}\\ \vec{K_{1}J_{1}}={\left[\begin{array}{ccc} 7.556 & 12.903 & -9.627 \end{array}\right]}^{T} \end{array}$$ (29)

Let the normal vector of the plane formed by the above two vectors be $\vec{F}$, then the vector relationship satisfies the following equation:


$$\{\begin{array}{c} \vec{F}\cdot \vec{G}=0\\ \vec{F}\cdot \vec{K_{1}L_{1}}=0 \end{array}$$ (30)

Solving the above equation yields $\vec{F}={\left[\begin{array}{ccc} -5.4604 & 10 & -17.6881 \end{array}\right]}^{T}$, similarly according to the right hand spiral rule yields $\vec{E}={\left[\begin{array}{ccc} 152.0904 & 10 & -41.2975 \end{array}\right]}^{T}$.

The position matrix TK1C of the coordinate system $K_{1XYZ}$ under the optical positioning system coordinate system $C_{XYZ}$ can be found by unitizing $\vec{E}$, $\vec{F}$ and $\vec{G}$, and determining the vector direction and associating the coordinate system and the origin:


TK1C=0.9631−0.2595−0.0709115.2030.06330.4753−0.8759123.550−0.2615−0.8407−0.47721038.5780001 (31)

(2) Obtaining the position information of the drill pin coordinate system in the optical positioning system coordinate system.

The target position information is converted to the robot base coordinate system by matrix transformation to obtain the target position matrix TK1B.Control the robot to the target point position, after reaching the predetermined position, the optical positioning system can obtain the position of the robot end optical position marker coordinate system in real time. Based on this position information, the position transformation matrix TO1C is calculated by Eq. (19).


TO1C1=0.86630.37970.3247132.461−0.0211−0.62160.7831159.3740.4991−0.6853−0.53041024.5810001 (32)

Convert Eq. (25) to obtain the position information TD2C of the drill pin coordinate system under the under the optical positioning system coordinate system.


TD2C=TO1C1TD2O1=0.9609−0.2074−0.1833114.18230.0530−0.51240.8571124.0254−0.2717−0.8333−0.48141039.38410001 (33)

(3) The positioning accuracy of the system is analyzed and measured using position deviation and pose deviation.

The position deviation is calculated using the Euclidean distance method, as shown in Eq. (34).


$$\Delta L=\sqrt{{\left(x_{i}-x_{j}\right)}^{2}+{\left(y_{i}-y_{j}\right)}^{2}+{\left(z_{i}-z_{j}\right)}^{2}}$$ (34)

The calculation of the pose deviation involves the rotation transformation, RDC is the rotation transformation matrix in the coordinate system of the implant handpiece drill pin relative to the coordinate system of the optical positioning system, and RKC is the rotation matrix in the coordinate system of the positioning block relative to the coordinate system of the optical positioning system, then:


ΔR=RDC(RKC)−1=r11r12r13r21r22r23r31r32r33 (35)

$$\begin{array}{l} \Delta R=R(Z,\Delta \alpha )R(Y,\Delta \beta )R(X,\Delta \gamma )\\ =\left[\begin{array}{ccc} c\Delta \alpha c\Delta \beta & s\Delta \alpha s\Delta \beta c\Delta \gamma -s\Delta \alpha c\Delta \gamma & c\Delta \alpha s\Delta \beta c\Delta \gamma +s\Delta \alpha s\Delta \gamma \\ s\Delta \alpha c\Delta \beta & s\Delta \alpha s\Delta \beta s\Delta \gamma -c\Delta \alpha c\Delta \gamma & s\Delta \alpha s\Delta \beta c\Delta \gamma -c\Delta \alpha s\Delta \gamma \\ -s\Delta \beta & c\Delta \beta s\Delta \gamma & c\Delta \beta c\Delta \gamma \end{array}\right] \end{array}$$ (36)

Combining Eq. (35) with Eq. (36) yields the pose deviation $\Delta \phi \text{=}{\left[\begin{array}{ccc} \Delta \gamma & \Delta \beta & \Delta \alpha \end{array}\right]}^{T}$, where: $\Delta \alpha \text{=}\arctan2\left(r_{21}/r_{11}\right)$, $\Delta \beta \text{=}\arcsin\left(-r_{31}\right)$, $\Delta \alpha \text{=}\arctan2\left(r_{32}/r_{33}\right)$, Δα ranges from $\left(-\pi ∼\pi \right)$, Δβ ranges from $\left(-\pi /2∼\pi /2\right)$ and Δγ ranges from $\left(-\pi ∼\pi \right)$.

Take the first set of experimental data as an example:


(ΔR)1=(RDC)1((RKC)−1)1=0.96320.0642−0.2610−0.25930.4782−0.8391−0.0711−0.8776−0.4742 (37)

The pose deviation is obtained according to the above method: $\Delta \alpha_{\text{1}}\text{=-2.0662}$, $\Delta \beta_{\text{1}}\text{=0.0712}$ and $\Delta \gamma_{1}=-0.2630$.

**Fig 19:**
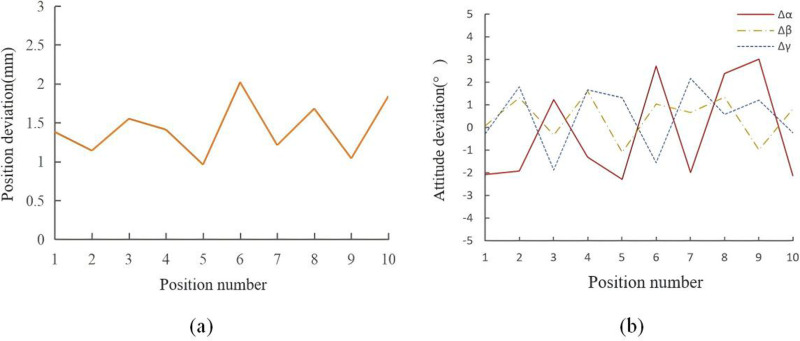
Position and pose deviation curves of each group of experiments: (a) Position deviation; (b) Pose deviation.

Based on the above methods and steps, data for the remaining nine groups were collected. This information is presented in Table 7. Using the data from the table, the pose of the target was determined and transformed into the robot coordinate system, which could then be used to guide the robot’s motion to the target position. The optical positioning system acquired the actual position and orientation of the drill needle, and the calculation results of the position and orientation deviation information are shown in Table 8. A position deviation curve is plotted, as shown in Fig 19, and the error is then analyzed.

**Table 7:** Optical spatial positioning block feature point position coordinate.

Serial number	Point *J*/mm	Point *K*/mm	Point *L*/mm
1	124.592,159.090,1041.283	115.203,123.550,1038.578	117.036,146.187,1050.910
2	134.651,163.571,1056.431	118.402,106.887,1046.474	121.367,156.681,1029.451
3	184.671,146.843,168.267	152.071,125.818,1073.186	136.541,135.415,1056.234
4	191.561,142.067,1094.325	173.134,137.358,1089.458	175.361,161.389,1091.264
5	198.341,150.233,1099.256	193.200,146.386,1102.274	203.065,156.423,1016.314
6	216.358,169.124,1135.624	208.385,154.470,1131.481	209,394,156.572,1135.641
7	234.267,168.125,1135.214	235.622,167.494,1131.982	236.501,171.361,1139.421
8	260.362,179.241,1179.264	259.934,177.954,1146.208	263.045,179.965,1046.314
9	281.167,191.204,1162.034	275.869,186.459,1158.146	267.168,196.051,1020.341
10	289.314,168.512,1171.267	289.889,194.486,1169.147	296.503,198.317,1173.323

**Table 8:** Optical spatial target point and measurement point postures and errors.

Serial number	Target point postures *x*_*K*_ / *mm* , *y*_*K*_ / *mm* , *z*_*K*_ / *mmα*_*K*_ / ° , *β*_*K*_ / ° , *γ*_*K*_ / °	Measure point postures *x*_*D*_ / *mm* , *y*_*D*_ / *mm* , *z*_*D*_ / *mmα*_*D*_ / ° , *β*_*D*_ / ° , *γ*_*D*_ / °	Position errors/mm	Pose errors/°
1		114.182,124.025,1039.384 3.1570,15.7658,−120.0152	1.38	−2.07, 0.07, −0.26
2	118.402,106.887,1046.474 7.9221,−9.9106,129.1650	119.141,105.991,1047.071 8.0261,−8.6610,131.3546	1.14	−1.93, 1.33, 1.79
3	152.071,125.818,1073.186 6.6685,−8.6106,128.3674	153.042,123.691,1074.196 6.8024,−9.6105,128.0426	1.55	1.24, −0.34, −1.88
4	173.134,137.358,1089.458 6.9655,−9.4647,128.3649	172.851,138.306,1088.342 6.8104,−9.6147,128.3648	1.41	−1.31, 1.61, 1.66
5	193.200,146.386,1102.274 4.1902,−4.1562,129.3547	194.024,143.304,1103.317 4.0591,−3.5064,129.0015	0.96	−2.28, −1.09, 1.33
6	208.385,154.470,1131.481 4.2964,−5.3924,129.1481	211.681,153.051,1133.497 4.6712,−6.0513,127.9450	2.02	2.71, 1.04, −1.55
7	235.622,167.494,1131.982 0.6912,1.4332,128.4081	236.241,169.141,1130.648 0.7213,1.6482,154.3610	1.21	−1.98, 0.67, 2.16
8	259.934,177.954,1146.208 −0.3554,4.8019,128.6459	261.381,179.315,1145.315 −0.6315,4.0129,138.4452	1.68	2.39, 1.34, 0.58
9	275.869,186.459,1158.146 0.6359,3.3305,128.0560	276.105,185.961,1157.261 0.5046,2.9406,131.0648	1.04	3.02, −0.99, 1.22
10	289.889,194.486,1169.147 1.1835,2.5691,128.2185	291.002,196.153,1171.165 0.9435,2.6904,130.5591	1.84	−2.11, 0.81, −0.22

From Fig 19(a), we observe that the average position error is 1.42 mm and the maximum position error is 2.02 mm. From Fig 19(b), we see that the average pose errors around the *x*, *y*, and *z* directions are 1.26°, 0.92°, and 2.1°, respectively, with the maximum pose error being 3.02°. Compared to existing research results, the overall positioning accuracy of the dental implant robot system constructed in this study is comparable to the current standard. However, the application of this system to implant surgery, which requires extremely high accuracy, necessitates an analysis of the errors for subsequent improvement.

The main factors contributing to large errors include the “Hand-Eye calibration,” which requires the optical positioning system and the robot to collect data simultaneously. Due to their inherent accuracy limitations, both systems can accumulate a certain degree of error during data collection, resulting in errors in the “Hand-Eye” calibration results. Additionally, the calibration accuracy of the “Six-point method” directly affects the final positioning accuracy of the system. Moreover, robot precision errors caused by uncertain factors in the robot’s manufacturing and assembly processes also impact the overall positioning precision of the system.

### 3.3 Accuracy analysis of the robot cavity preparation

#### 3.3.1 Error measurement.

**Fig 20:**
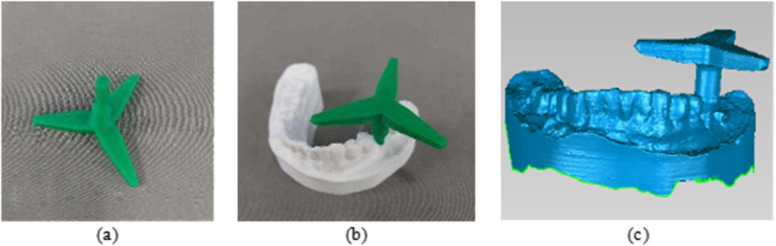
Digital model acquisition of assembly model surface: (a) Registration block; (b) Assembly model; (c) Digital Model.

In this study, we designed and fabricated registration block and combined them with an optical scanner to measure errors in experimental results, as opposed to the traditional method of CT scanning. The registration block was designed and 3D printed to ensure the axial hole fit with the model-prepared cavities in sequence. The different assemblies were then scanned using the Bluefield Medical Runyes Dental Optical Scanner, and the acquired digital model was saved in STL format, as shown in Fig 20.

**Fig 21:**
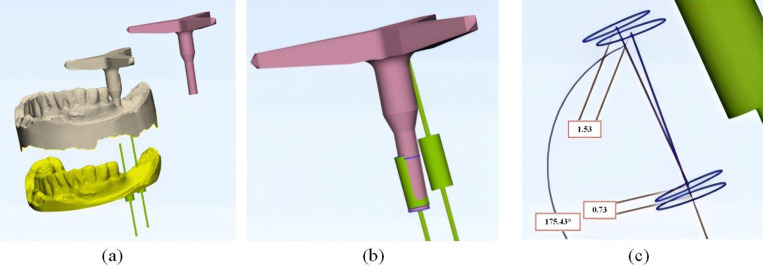
Model registration and error measurement: (a) Model registration; (b) Model relative position after registration; (c) Error measurement.

The registration block, the implant scheme model, and the STL format file of the acquired assembly were imported into the 3-matic software together. The tooth-mucosa model was used as the base model, while the rest of the models were treated as floating models. After completing the registration, the tooth-mucosa model and the assembly model were hidden. The error between the actual cavity (the cylinder at the end of the registration block) and the virtual implant was measured using the software’s measurement tool, as shown in Fig 21. The cavity preparation errors for the Experimental Group (Robotic Group) and the Control Group (Manual Group)were obtained using the method described above.

#### 3.3.2 Results and discussion.

(1) Statistical results of error data

After completing the experiments for both the Experimental Group (Robotic Group) and the Control Group (Manual Group), which involved 5 sets of experiments for each group (a total of 10 cavities), the obtained data error (Table 9), including top deviation, root deviation, and angle deviation, were imported into SPSS for statistical analysis.

**Table 9:** Error data of the robot-prepared cavities and manual cavities.

Groups	Numbers	Cave number	Top deviation/mm	Root deviation/mm	Angular deviation/°
Experimental Group (Robot Group)	1	36	1.13	0.73	4.57
		37	0.94	1.46	4.31
	2	36	1.02	2.33	3.58
		37	1.87	1.74	2.95
	3	36	1.12	1.94	4.02
		37	0.92	1.56	4.28
	4	36	1.08	1.60	4.13
		37	1.34	1.46	3.99
	5	36	1.10	0.87	4.55
		37	1.40	1.55	4.12
Control Group (Manual Group)	1	36	1.32	2.45	5.10
		37	1.65	2.87	5.52
	2	36	1.45	2.61	5.25
		37	1.73	2.91	5.63
	3	36	1.39	2.50	5.15
		37	1.80	3.05	5.75
	4	36	1.50	2.70	5.35
		37	1.82	3.12	5.80
	5	36	1.48	2.60	5.30
		37	1.76	3.00	5.65

The normality test (Shapiro-Wilk test) was applied to check the distribution of the error data, and the results confirmed that all error data followed a normal distribution. Therefore, independent sample t-tests were used to compare the differences between the two groups. Table 10 shows the statistical results of the error data for both the experimental and control groups, presenting the mean values ± standard deviations.

**Table 10:** Statistical results of cavity preparation errors between the Experimental Group and the Control Group.

Groups	Top deviation/mm	Root deviation/mm	Angular deviation/°
Experimental Group (Robot Group)	1.09 ± 0.37	1.49 ± 0.57	4.17 ± 0.28
Control Group (Manual Group)	1.43 ± 0.06	2.57 ± 0.10	5.23 ± 0.10

The independent sample t-test results showed that there were no statistically significant differences between the two groups for top deviation and root deviation (*P* > 0.05). However, for angle deviation, the experimental group (robotic group) exhibited significantly lower values compared to the control group (*P* < 0.05).From these results, it can be observed that the robotic group and the traditional surgery group had similar values for top deviation and root deviation, with no significant differences observed. However, the robotic group demonstrated superior directional control precision in terms of angle deviation.

(2) Results analysis and discussion
  (a) Top and root deviations

The experimental results indicate that the top deviation in the robot group (1.09 ± 0.37 mm) is similar to that in the control group (1.43 ± 0.06 mm). The root deviation and angular deviation are also within reasonable ranges. This suggests that the robot system’s positioning capability is close to or has reached the level of an experienced clinical surgeon, meeting or approximating the precision requirements of clinical implant procedures. This outcome may be related to the pre-positioning role of the personalized guide plate mapping device, which provides highly precise reference points for the initial positioning and feeding direction. As a result, both the robot and the surgeon can perform the procedure with a high level of precision. Additionally, the stiffness differences between the 3D printed mandibular model and actual clinical bone tissue may make it easier for both manual and robotic operations to achieve ideal precision in depth and displacement control.

(b) Angle deviation

Regarding angle deviation, the robot group’s average deviation was 3.93 ± 0.55°, significantly lower than the control group’s 4.15 ± 0.85°. This indicates that the robot system has a clear advantage in direction control. Factors influencing angle deviation include: the robot system’s precise pose control and pre-planned path, which effectively avoid hand tremors and operational errors commonly encountered in manual procedures, thus improving precision. Although the control group’s procedure was performed by an experienced surgeon, manual drilling can still be affected by hand vibrations and operational deviations, resulting in slightly poorer angle control.

(3) Sources of error and improvement directions

Although the robot group showed better angle control, there is still room for further improvement in overall error. The main sources of error include: slight deformations in the 3D printed guide plate mapping device and model, which may introduce minor errors during initial scheme registration. This could be reduced by improving printing accuracy, using higher-quality materials, or optimizing the guide plate design. The calibration precision of the robot’s hand-eye system directly affects the accuracy of pose conversion. Future improvements could focus on optimizing the calibration algorithm and the design of calibration fixtures to reduce system errors. Additionally, the experiment was conducted on a resin model, which has significant differences in hardness and stiffness compared to actual bone tissue. Future studies may use bone-like materials or animal bone models to better reflect clinical surgical outcomes.

(4) Comparison with other studies

The reported accuracy of computer-assisted implant systems and robotic implants (such as Schneider [27], Pettersson [28], etc.) is similar to the results of this study, with some differences in angle control. Compared to previous literature, this study’s results show that the top and root errors are already close to clinically acceptable ranges, while angle accuracy shows a certain advantage. A comparison with existing research validates the robot system proposed in this study, which achieves comparable or even superior accuracy compared to traditional methods.

(5) Clinical translation and future applications

The experimental results confirm the feasibility of the dental implant robotic system in cavity preparation accuracy, particularly demonstrating higher stability and repeatability in angle control. This provides favorable technical evidence for future clinical translation. Through further optimization of guide plate designs, improvement of calibration precision, and validation in environments closer to clinical conditions, the dental implant robotic system has the potential to reduce implant errors, improve surgical quality, and lower the incidence of complications and secondary surgeries in clinical settings.

## 4. Conclusions

In this study, we developed a comprehensive oral implant robot system, which includes innovations in the design and fabrication of a personalized dental implant guide mapping device, spatial information registration, and cavity preparation experiments. These advancements significantly enhance the precision, safety, and predictability of oral implant surgery, offering innovative technical solutions to improve surgical outcomes. The specific contributions of this study are outlined as follows:

(1) Construction of the Oral Implant Robot System

To address the limitations of traditional cavity preparation methods, we developed an efficient and precise oral implant robot system by integrating digital implant guides with optical positioning navigation technology. This approach improves the accuracy and efficiency of cavity preparation, offering a robust solution to current challenges in dental implant surgeries.

(2) Design and Validation of the Personalized Dental Implant Guide Mapping Device

Utilizing CBCT image data and a digital model of the tooth-mucosa of an artificial jaw, we created a personalized implant plan using Mimics software. The implant guide mapping device was designed using Geomagic, 3-matic, and SolidWorks software and fabricated through 3D printing. Optical scanning and precise registration verification confirmed that the guide plate exhibits excellent guiding accuracy and stable repeatability, effectively meeting the high-precision requirements of oral implant surgery. The successful integration of these technologies demonstrates the viability of personalized solutions for implant procedures.

(3) Spatial Information Registration of the Oral Implant Robot System

We thoroughly analyzed the coordinate systems within the surgical robot system and their transformation relationships. Through accurate hand-eye calibration and end-tool calibration, we unified the system’s coordinate systems. Experimental verification of the system’s overall positioning accuracy confirmed the feasibility and precision of our spatial information registration method, ensuring reliable operation for the dental implant robot system. This method is crucial for achieving high-precision outcomes in surgery.

(4) Experiment on Cavity Preparation Using the Dental Implant Robot

This study compared the performance of the robotic system with traditional surgical methods. Results showed that the robotic system significantly outperformed manual methods in terms of angular control precision, with much lower angular deviations (4.17 ± 0.28° vs. 5.23 ± 0.10°). However, no significant differences were observed in top deviation (1.09 ± 0.37 mm vs. 1.43 ± 0.06 mm) and root deviation (1.49 ± 0.57 mm vs. 2.57 ± 0.10 mm) between the two groups. The robotic system demonstrated high precision in directional control, effectively reducing human error, particularly in angular control. Despite these successes, further improvements in overall accuracy are necessary, particularly in model and template printing precision, as well as optimization of calibration methods to minimize errors.

This research presents a novel and reproducible methodology for evaluating the precision of dental implant guide plates and robotic positioning systems. By demonstrating the potential of robotic assistance to enhance surgical precision and outcomes in dental implantology, this study provides valuable insights for future clinical applications and technological advancements in the field. The continued development of these technologies has the potential to revolutionize the way oral implant surgeries are performed, offering safer, more predictable, and highly precise procedures.

## Supporting information

S1. Text Data sheet. (DOCX)
