# Supplementary material for: Development and precision evaluation of a robotic system for oral implant surgery using personalized digital guides and optical spatial positioning technology
Source: PLoS One. 2025 Apr 29;20(4):e0319054. doi: 10.1371/journal.pone.0319054 (PMC12040283; doi:10.1371/journal.pone.0319054)
Supplement: S1. Text — (DOCX) [file pone.0319054.s001.docx]

Table 3 Position information of each point under optical positioning system

| Missing tooth position | Initial drilling point *P*/mm | | | Guide cylinder end point *Q*/mm | | |
| --- | --- | --- | --- | --- | --- | --- |
|  | $(P^{C})_{x}$ | $(P^{C})_{y}$ | $(P^{C})_{z}$ | $(Q^{C})_{x}$ | $(Q^{C})_{y}$ | $(Q^{C})_{z}$ |
| 36 | 131.115 | 266.676 | 1191.352 | 130.78 | 250.835 | 1188.936 |
| 37 | 131.548 | 263.835 | 1199.05 | 131.573 | 247.701 | 1196.093 |

Table 4 Position information of each point in the coordinate system $O_{2xyz}$

| Missing tooth position | Initial drilling point *P*/mm | | | Guide cylinder end point *Q*/mm | | |
| --- | --- | --- | --- | --- | --- | --- |
|  | $(P^{O_{2}})_{x}$ | $(P^{O_{2}})_{y}$ | $(P^{O_{2}})_{z}$ | $(Q^{O_{2}})_{x}$ | $(Q^{O_{2}})_{y}$ | $(Q^{O_{2}})_{z}$ |
| 36 | 32.4293 | -25.8123 | -57.1797 | 45.4826 | -18.3781 | -62.7684 |
| 37 | 34.7653 | -30.9019 | -63.1924 | 48.2806 | -23.1591 | -68.3345 |

**Table 5.** Distance and angle error between the axis of the guide plate and virtual implant

| **Number of experiments** | **Tooth number** | **Axis distance error /mm** | **Axis angle error /°** |
| --- | --- | --- | --- |
| 1 | 36 | 0.84 | 2.36 |
|  | 37 | 0.74 | 2.70 |
| 2 | 36 | 0.83 | 2.37 |
|  | 37 | 0.73 | 2.71 |
| 3 | 36 | 0.85 | 2.34 |
|  | 37 | 0.74 | 2.70 |
| 4 | 36 | 0.83 | 2.36 |
|  | 37 | 0.72 | 2.71 |
| 5 | 36 | 0.86 | 2.33 |
|  | 37 | 0.73 | 2.69 |

**Table 6.** Position information of each feature point of positioning block under optical positioning system

| **Feature points** | **Feature point coordinates /mm** |
| --- | --- |
| $J_{1}$ | 124.592, 159.090, 1041.283 |
| $K_{1}$ | 115.203, 123.550, 1038.578 |
| $L_{1}$ | 117.036, 146.187, 1050.910 |

**Table 7.** Optical spatial positioning block feature point position coordinate

| **Serial number** | **Point *J*/mm** | **Point *K*/mm** | **Point *L*/mm** |
| --- | --- | --- | --- |
| 1 | 124.592,159.090,1041.283 | 115.203,123.550,1038.578 | 117.036,146.187,1050.910 |
| 2 | 134.651,163.571,1056.431 | 118.402,106.887,1046.474 | 121.367,156.681,1029.451 |
| 3 | 184.671,146.843,168.267 | 152.071,125.818,1073.186 | 136.541,135.415,1056.234 |
| 4 | 191.561,142.067,1094.325 | 173.134,137.358,1089.458 | 175.361,161.389,1091.264 |
| 5 | 198.341,150.233,1099.256 | 193.200,146.386,1102.274 | 203.065,156.423,1016.314 |
| 6 | 216.358,169.124,1135.624 | 208.385,154.470,1131.481 | 209,394,156.572,1135.641 |
| 7 | 234.267,168.125,1135.214 | 235.622,167.494,1131.982 | 236.501,171.361,1139.421 |
| 8 | 260.362,179.241,1179.264 | 259.934,177.954,1146.208 | 263.045,179.965,1046.314 |
| 9 | 281.167,191.204,1162.034 | 275.869,186.459,1158.146 | 267.168,196.051,1020.341 |
| 10 | 289.314,168.512,1171.267 | 289.889,194.486,1169.147 | 296.503,198.317,1173.323 |

**Table 8.** Optical spatial target point and measurement point postures and errors

| **Serial number** | **Target point postures**  **$\begin{matrix} x_{K}/\mathrm{mm},y_{K}/\mathrm{mm},z_{K}/\mathrm{mm} \\ \alpha_{K}/^{\circ},\beta_{K}/^{\circ},\gamma_{K}/^{\circ} \end{matrix}$** | **Measure point postures**  **$\begin{matrix} x_{D}/\mathrm{mm},y_{D}/\mathrm{mm},z_{D}/\mathrm{mm} \\ \alpha_{D}/^{\circ},\beta_{D}/^{\circ},\gamma_{D}/^{\circ} \end{matrix}$** | **Position errors/mm** | **Pose errors/**° |
| --- | --- | --- | --- | --- |
| 1 | 115.203,123.550,1038.578  3.7604,15.1368,-119.5802 | 114.182,124.025,1039.384  3.1570,15.7658,-120.0152 | 1.38 | -2.07，0.07，-0.26 |
| 2 | 118.402,106.887,1046.474  7.9221,-9.9106,129.1650 | 119.141,105.991,1047.071  8.0261,-8.6610,131.3546 | 1.14 | -1.93，1.33，1.79 |
| 3 | 152.071,125.818,1073.186  6.6685,-8.6106,128.3674 | 153.042,123.691,1074.196  6.8024,-9.6105,128.0426 | 1.55 | 1.24，-0.34，-1.88 |
| 4 | 173.134,137.358,1089.458  6.9655,-9.4647,128.3649 | 172.851,138.306,1088.342  6.8104,-9.6147,128.3648 | 1.41 | -1.31，1.61，1.66 |
| 5 | 193.200,146.386,1102.274  4.1902,-4.1562,129.3547 | 194.024,143.304,1103.317  4.0591,-3.5064,129.0015 | 0.96 | -2.28，-1.09，1.33 |
| 6 | 208.385,154.470,1131.481  4.2964,-5.3924,129.1481 | 211.681,153.051,1133.497  4.6712,-6.0513,127.9450 | 2.02 | 2.71，1.04，-1.55 |
| 7 | 235.622,167.494,1131.982  0.6912,1.4332,128.4081 | 236.241,169.141,1130.648  0.7213,1.6482,154.3610 | 1.21 | -1.98，0.67，2.16 |
| 8 | 259.934,177.954,1146.208  -0.3554,4.8019,128.6459 | 261.381,179.315,1145.315  -0.6315,4.0129,138.4452 | 1.68 | 2.39，1.34，0.58 |
| 9 | 275.869,186.459,1158.146  0.6359,3.3305,128.0560 | 276.105,185.961,1157.261  0.5046,2.9406,131.0648 | 1.04 | 3.02，-0.99，1.22 |
| 10 | 289.889,194.486,1169.147  1.1835,2.5691,128.2185 | 291.002,196.153,1171.165  0.9435,2.6904,130.5591 | 1.84 | -2.11，0.81，-0.22 |

**Table 9**. Error data of the robot-prepared cavities and manual cavities

| **Groups** | **Numbers** | **Cave number** | **Top deviation/mm** | **Root deviation/mm** | **Angular deviation/°** |
| --- | --- | --- | --- | --- | --- |
| Experimental Group  (Robot Group) | **1** | **36** | 1.13 | 0.73 | 4.57 |
|  |  | **37** | 0.94 | 1.46 | 4.31 |
|  | **2** | **36** | 1.02 | 2.33 | 3.58 |
|  |  | **37** | 1.87 | 1.74 | 2.95 |
|  | **3** | **36** | 1.12 | 1.94 | 4.02 |
|  |  | **37** | 0.92 | 1.56 | 4.28 |
|  | **4** | **36** | 1.08 | 1.60 | 4.13 |
|  |  | **37** | 1.34 | 1.46 | 3.99 |
|  | **5** | **36** | 1.10 | 0.87 | 4.55 |
|  |  | **37** | 1.40 | 1.55 | 4.12 |
| Control Group  (Manual Group) | **1** | **36** | 1.32 | 2.45 | 5.10 |
|  |  | **37** | 1.65 | 2.87 | 5.52 |
|  | **2** | **36** | 1.45 | 2.61 | 5.25 |
|  |  | **37** | 1.73 | 2.91 | 5.63 |
|  | **3** | **36** | 1.39 | 2.50 | 5.15 |
|  |  | **37** | 1.80 | 3.05 | 5.75 |
|  | **4** | **36** | 1.50 | 2.70 | 5.35 |
|  |  | **37** | 1.82 | 3.12 | 5.80 |
|  | **5** | **36** | 1.48 | 2.60 | 5.30 |
|  |  | **37** | 1.76 | 3.00 | 5.65 |

**Table 10.** Statistical results of cavity preparation errors between the Experimental Group and the Control Group

| **Groups** | **Top deviation/mm** | **Root deviation/mm** | **Angular deviation/°** |
| --- | --- | --- | --- |
| Experimental Group (Robot Group) | 1.09 ± 0.37 | 1.49 ± 0.57 | 4.17 ± 0.28 |
| Control Group (Manual Group) | 1.43 ± 0.06 | 2.57 ± 0.10 | 5.23 ± 0.10 |
